# Gut-associated cGMP mediates colitis and dysbiosis in a mouse model of an activating mutation in *GUCY2C*

**DOI:** 10.1084/jem.20210479

**Published:** 2021-09-21

**Authors:** Vishwas Mishra, Avipsa Bose, Shashi Kiran, Sanghita Banerjee, Idrees A. Shah, Pooja Chaukimath, Mudasir M. Reshi, Swarna Srinivas, Anaxee Barman, Sandhya S. Visweswariah

**Affiliations:** Department of Molecular Reproduction, Development and Genetics, Indian Institute of Science, Bengaluru, India

## Abstract

Activating mutations in receptor guanylyl cyclase C (GC-C), the target of gastrointestinal peptide hormones guanylin and uroguanylin, and bacterial heat-stable enterotoxins cause early-onset diarrhea and chronic inflammatory bowel disease (IBD). GC-C regulates ion and fluid secretion in the gut via cGMP production and activation of cGMP-dependent protein kinase II. We characterize a novel mouse model harboring an activating mutation in *Gucy2c* equivalent to that seen in an affected Norwegian family. Mutant mice demonstrated elevated intestinal cGMP levels and enhanced fecal water and sodium content. Basal and linaclotide-mediated small intestinal transit was higher in mutant mice, and they were more susceptible to DSS-induced colitis. Fecal microbiome and gene expression analyses of colonic tissue revealed dysbiosis, up-regulation of IFN-stimulated genes, and misregulation of genes associated with human IBD and animal models of colitis. This novel mouse model thus provides molecular insights into the multiple roles of intestinal epithelial cell cGMP, which culminate in dysbiosis and the induction of inflammation in the gut.

## Teaser

A novel preclinical mouse model harboring an activating mutation in *Gucy2c* demonstrates features seen in patients with familial *GUCY2C* diarrhea syndrome. Fecal microbiome changes and colonic gene expression identify cell intrinsic and extrinsic roles of cGMP in gut inflammation.

## Graphical Abstract

**Figure:**
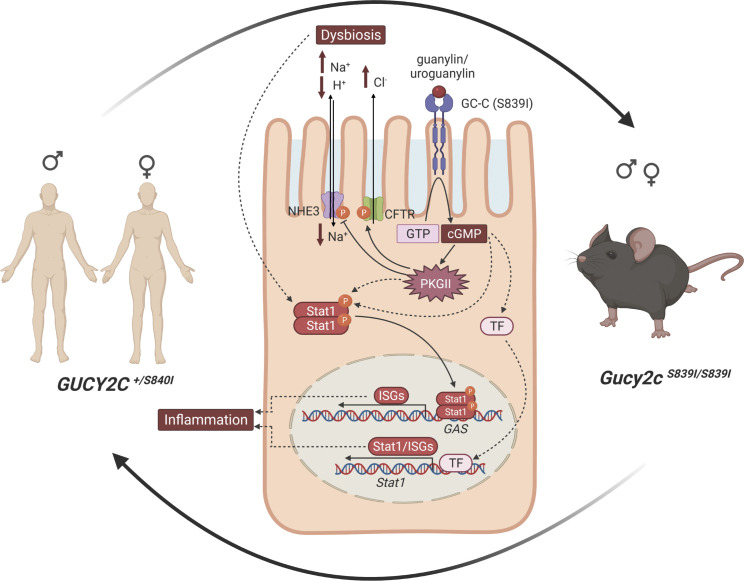

## Introduction

Monogenic intestinal epithelium defects contributing to pediatric inflammatory bowel disease (IBD) have been described and are not readily amenable to current treatment regimens (Leung and Muise, 2018; Nambu and Muise, 2021). Among the genes associated with very early–onset IBD are mutations in the receptor guanylyl cyclase C gene (*GUCY2C*; Bose et al., 2020; Crowley et al., 2020). The receptor encoded by this gene, guanylyl cyclase C (GC-C), is the target of the gastrointestinal hormones guanylin (encoded by *GUCA2A*) and uroguanylin (encoded by *GUCA2B*; Arshad and Visweswariah, 2012; Basu et al., 2010). GC-C is predominantly expressed along the gastrointestinal tract, where it regulates fluid and ion transport across the intestinal epithelium (Waldman and Camilleri, 2018). Ligand binding to GC-C results in elevated 3′5′-cyclic guanosine monophosphate (cGMP) levels in the intestinal cell and activation of cGMP-dependent protein kinase II (PKGII; Lohmann et al., 1997). PKGII phosphorylates the cystic fibrosis transmembrane conductance regulator (CFTR) and the sodium-hydrogen exchanger, NHE3 (Chen et al., 2015; Golin-Bisello et al., 2005). Phosphorylation of CFTR increases secretion of chloride and bicarbonate ions, while phosphorylation of NHE3 inhibits sodium uptake by the intestinal epithelial cell (IEC; Chen et al., 2015). The ensuing osmotic imbalance across the IEC causes efflux of water from the cell required for mucus hydration and passage of the bolus of food along the gut (Arshad and Visweswariah, 2013).

Familial *GUCY2C* diarrhea syndrome (FGDS) was first described in a Norwegian family where >30 individuals reported diarrhea of varying severity from infancy onward (Fiskerstrand et al., 2012). The autosomal dominant mutation mapped to *GUCY2C* resulted in a change of Ser840 to isoleucine, present in the guanylyl cyclase domain. The mutation resulted in hyperactivation of GC-C whereby the mutant receptor elicited greater levels of cGMP on stimulation with ligands. These elevated cGMP levels presumably overactivated downstream signaling, resulting in increased fluid and ion secretion and diarrhea (Fiskerstrand et al., 2012). Subsequently, we characterized an additional four activating mutations in unrelated children in Europe, who also presented with severe and debilitating diarrhea, detectable in utero as a greatly distended abdomen in the fetus (Müller et al., 2016). The mutations (Lys507Glu, Leu775Pro, Arg792Ser, and Asn850Asp) were present in different domains of the receptor, including the kinase-homology domain, the linker region, and the catalytic domain (Bose et al., 2020; Mishra et al., 2018). Patients suffering from FGDS and children with de novo mutations in *GUCY2C* present with Crohn’s disease (CD)–like symptoms and colitis in addition to diarrhea (Fiskerstrand et al., 2012; Müller et al., 2016).

GC-C is the target of bacterial heat-stable enterotoxins (STs) produced by enterotoxigenic *Escherichia coli*, one of the major causes of watery diarrhea in developing countries (Schulz et al., 1990). The Food and Drug Administration–approved drugs linaclotide and plecanatide, which are used to treat constipation, comprise the cysteine-rich core sequence of ST or uroguanylin, respectively, and activate GC-C to induce cGMP production (Shah et al., 2018). However, no drugs are available to alleviate diarrhea mediated by GC-C, though molecules that may inhibit proteins downstream of GC-C show promise (Bijvelds et al., 2018).

Knockout mice for GC-C have been studied for several years (Schulz et al., 1997). While they show no apparent signs of constipation, reports indicate that they demonstrate neurological symptoms (Brierley, 2012; Mann et al., 2019), and suppression of uroguanylin-mediated GC-C signaling in mice results in obesity (Valentino et al., 2011). We have demonstrated that GC-C^−/−^ mice are more susceptible to oral *Salmonella enterica* enterica serovar Typhimurium infection (Majumdar et al., 2018). Inactivating mutations have been reported in *GUCY2C*, with infants presenting with meconium ileus at birth. These children were the outcome of consanguinity, with mutations present in a homozygous state (Romi et al., 2012; Smith et al., 2015; Woods et al., 2019).

The lack of models for diarrheal disease mediated by hyperactive GC-C to investigate the link between GC-C, cGMP, and gut inflammation prompted us to develop a mutant mouse harboring a mutation in *Gucy2c*, equivalent to the S840I mutation found in the Norwegian family. Here, we validate this model in terms of increased frequency of passing watery feces, regulation of Cftr and Nhe3 activity in vivo, and enhanced susceptibility of these mice to colitis. Analysis of the fecal microbiome in mutant mice and global colonic gene expression provided the underlying explanation for inflammation observed in patients harboring activating mutations of GC-C. Therefore, this mutant mouse serves as a preclinical model for *GUCY2C*-mediated secretory diarrhea associated with IBD and the more prevalent infectious diarrheal illness caused by GC-C activation.

## Results

### Biochemical characterization of the S839I mutation in mouse GC-C

**Figure 1.**
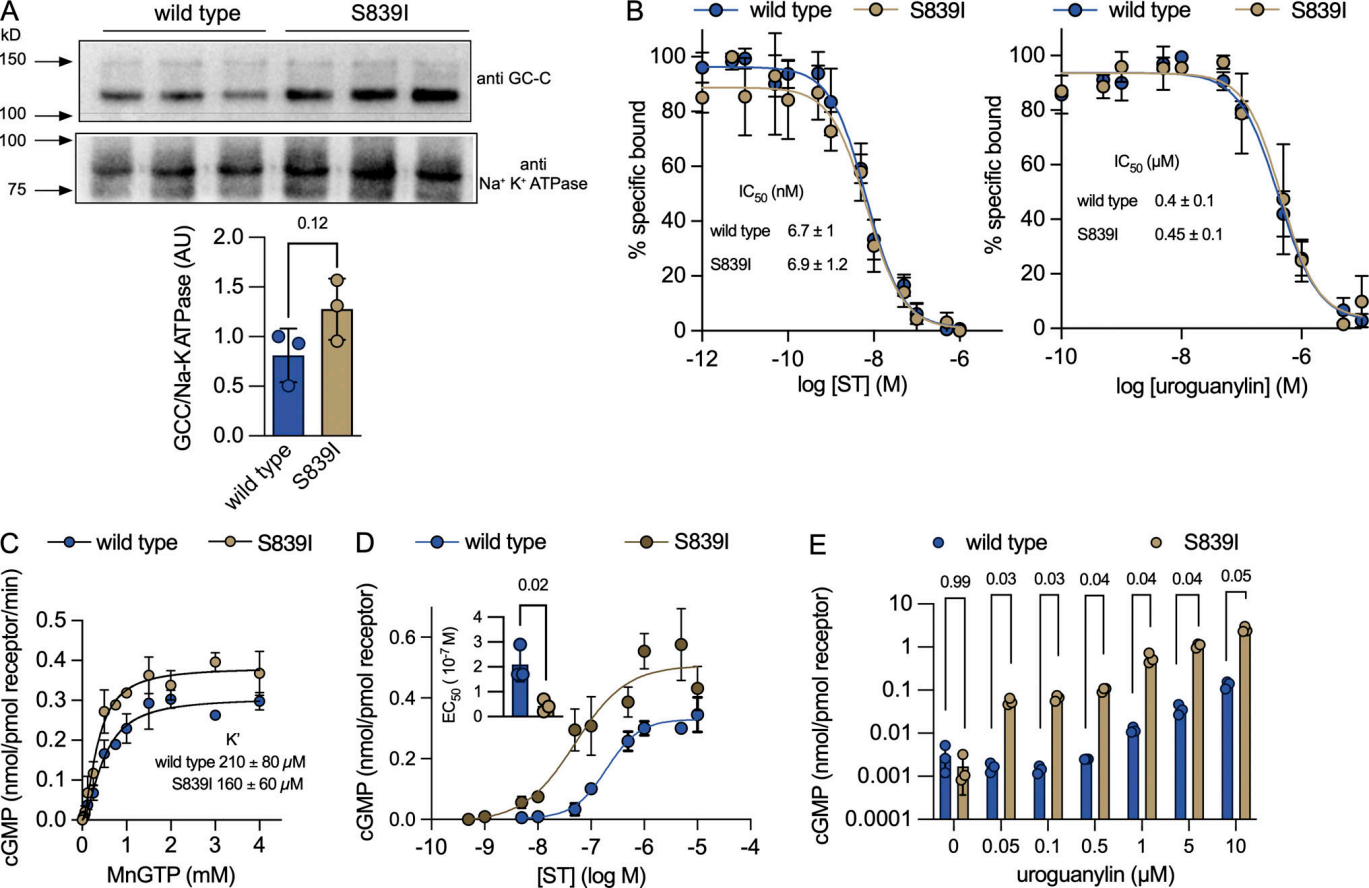
**Biochemical characterization of the p.S839I murine GC-C. (A)** Western blot of membranes prepared from three independent cultures of HEK293E cells stably expressing either wild type or S839I mutant receptor. Na^+^/K^+^ ATPase was used as loading control. Densitometric analysis was performed, and P value shown is from unpaired, two-tailed *t* test. Experiments were repeated twice and values shown are the mean ± SD. **(B)** Binding of ^125^I-labeled ST peptide in the presence of varying concentrations of ST (left) or mouse uroguanylin (right). Inset values represent the mean IC_50_ ± SD of determinations of experiments repeated thrice. **(C)** Catalytic activity of wild type and S839I mutant GC-C using MnGTP (guanosine triphosphate) as substrate. Inset values represent the mean K' ± SD of experiments repeated thrice and values shown are the mean ± SD. **(D)** HEK293E cells expressing either wild type or mutant GC-C were treated with the indicated concentrations of ST and cGMP measured by radioimmunoassay. Cyclic GMP produced was normalized to receptor concentrations (see Materials and methods). Values represent mean ± SD from experiments performed thrice. Inset: the EC_50_ values were calculated from independent experiments, and P values shown were obtained by an unpaired, two-tailed *t* test with Welch’s correction. **(E)** HEK293E cells expressing either wild type or mutant GC-C were treated with medium alone or uroguanylin and cGMP produced estimated by radioimmunoassay. Values represent mean ± SD from experiments performed twice with triplicate determinations at each concentration. P values shown are from unpaired, two-tailed *t* tests with Welch’s correction. ATPase, adenosine triphosphatase.

The S840 residue in human GC-C is equivalent to S839 in mouse GC-C due to the absence of M701 in mouse GC-C (Fiskerstrand et al., 2012). We generated two stable HEK293E cell lines expressing either mutant (S839I) or wild type mouse GC-C. No significant difference in expression of the two forms of GC-C in the cell lines was detected (Fig. 1 A). Both receptor forms bound ST peptide and mouse uroguanylin with similar affinities (Fig. 1 B) and showed similar in vitro guanylyl cyclase activity (Fig. 1 C). However, the mutant receptor showed enhanced ligand-mediated cGMP production (Fig. 1, D and E), as seen with the S840I mutation in human GC-C (Fiskerstrand et al., 2012). Importantly, the EC_50_ (half-maximal effective concentration) of ST for activation of the mutant mouse receptor was significantly lower than that of the wild type receptor (Fig. 1 D).

**Figure S1.**
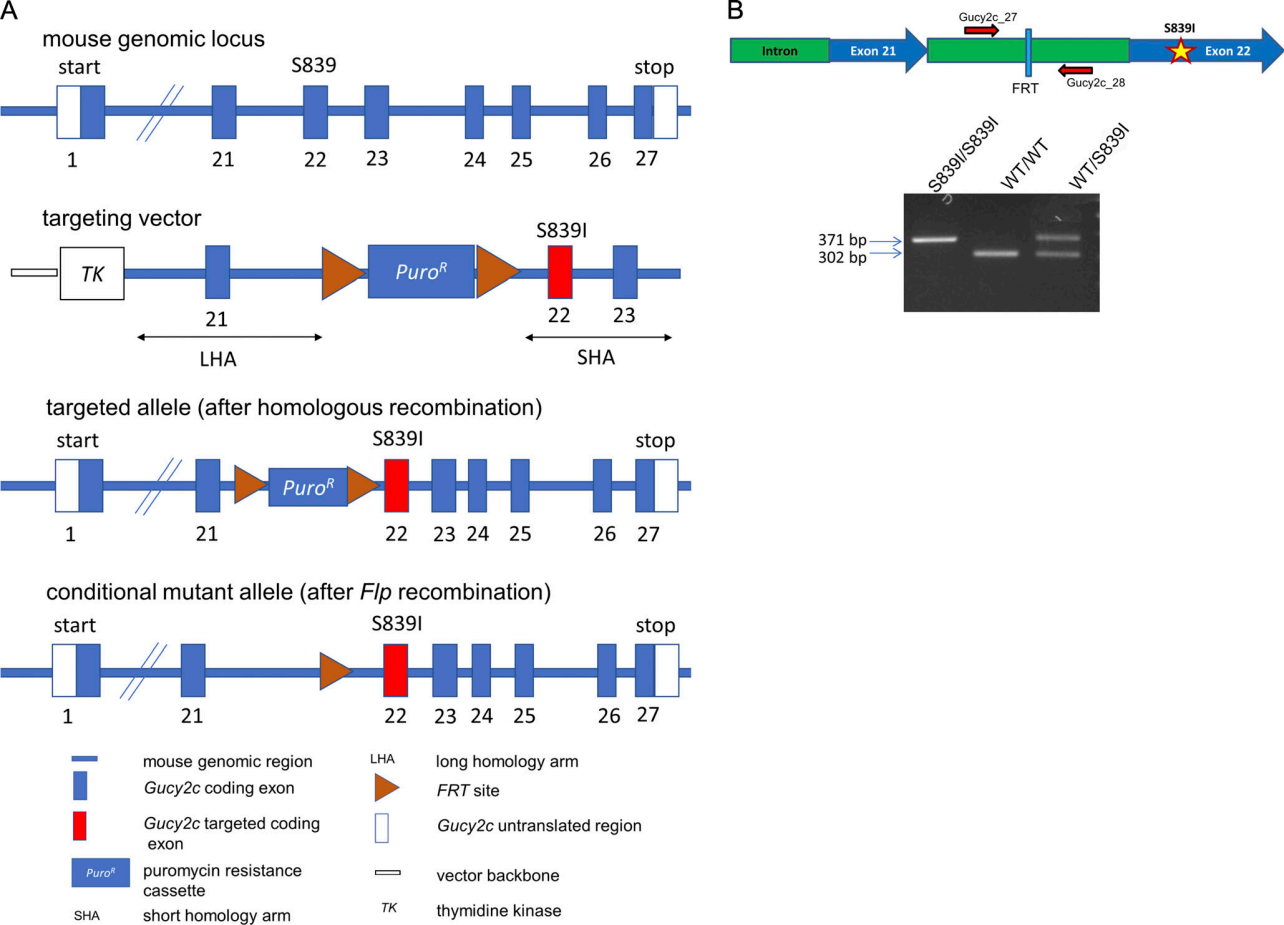
**Generation of mutant mouse with p.S839I mutation in GC-C. (A)** Schematic representation illustrating the principle behind generation of the mice with p.S839I mutation in GC-C. The S839 amino acid is present in exon 22 of the *Gucy2c* locus. Targeting vectors were generated using BAC clones from C57BL/6J RPCIB-731 BAC library and transfected into Taconic Artemis C57BL/6N Tac ES cell line. The homologous recombinant clones were isolated using puromycin selection. The constitutive KI mutation was obtained after in vivo FLP-mediated removal of puromycin selection marker. **(B)** Top panel depicts a schematic representation of the intronic region between exons 21 and 22 in the *Gucy2c* locus with FRT cassette and annealing sites for primers used to PCR amplify the region of interest for the purpose of genotyping the mice. Image not drawn to scale. Bottom panel is a representative agarose gel image used for genotyping the mice. Genomic DNA was isolated from tail snips of mice, and PCR amplification of the region encompassing the FRT site was performed. PCR amplification in mutant allele resulted in a product that is 69 bp longer than wild type allele product displaying the insertion of the FRT cassette.

**Figure S2.**
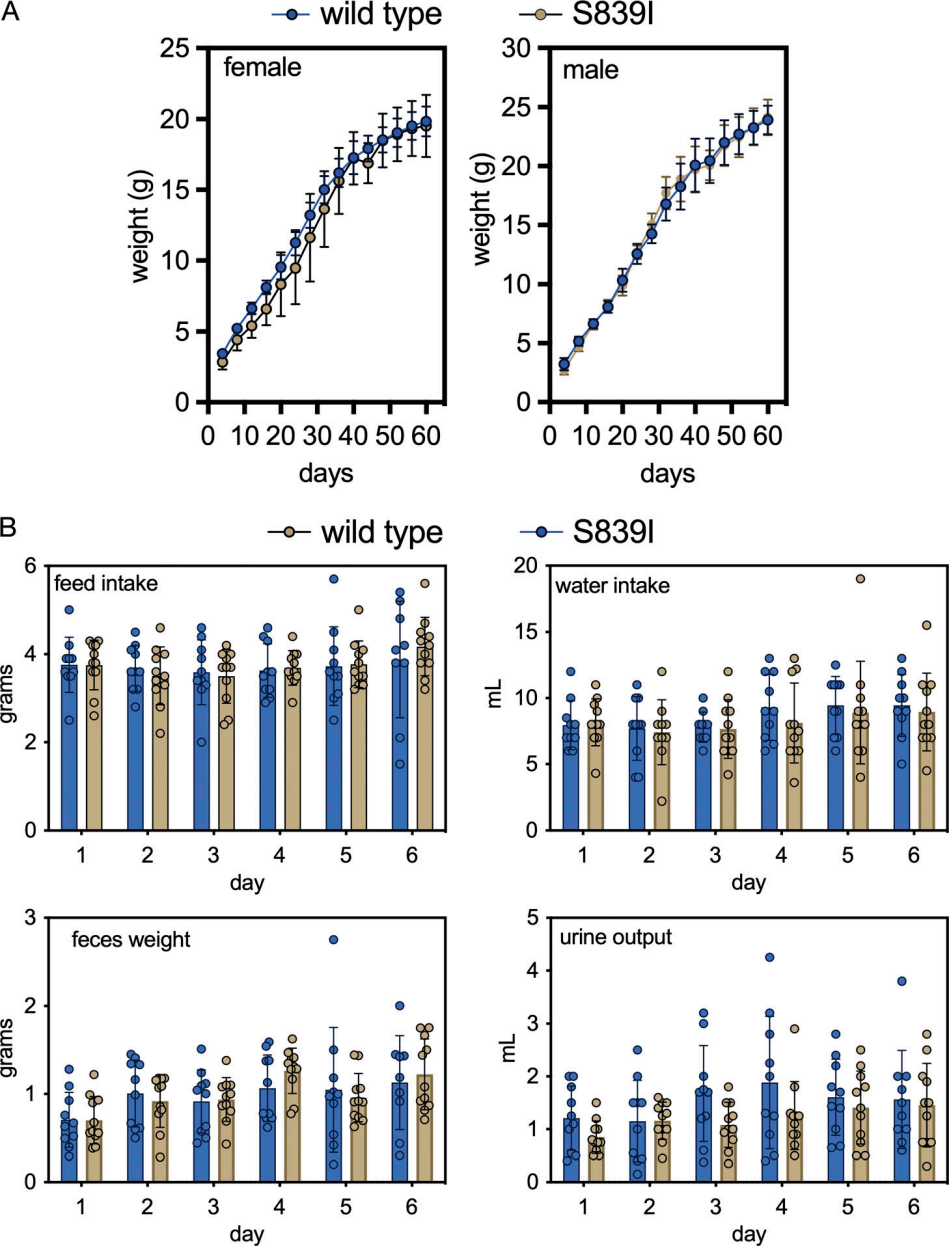
**Weight gain and food intake of S839I mice. (A)** Mice were weighed from day 4 after parturition. Weight gain is shown from 13 mice from multiple litters. Data shown are mean ± SD. **(B)** Mice of 40–45 d of age were placed in metabolic cages, and feed and water consumed was measured daily for 6 d. Feces were collected over 24 h, and volume of urine output was measured over the time mice were in metabolic cages. Data shown are mean ± SD.

We proceeded to generate a mutant mouse harboring a point mutation in *Gucy2c* at position S839 (Fig. S1 and Materials and methods). We outcrossed mutant mice against wild type C57/BL6N Tac mice for at least 10 generations before use. No significant difference in weight gain was observed between wild type and mutant mice. Food, water intake, and fecal and urine output were similar as measured from data collected from mice housed individually in metabolic cages (Fig. S2). We henceforth refer to these mutant mice as S839I mice.

### Elevated intestinal epithelial cGMP levels and features of bowel dysfunction in S839I mice

**Figure 2.**
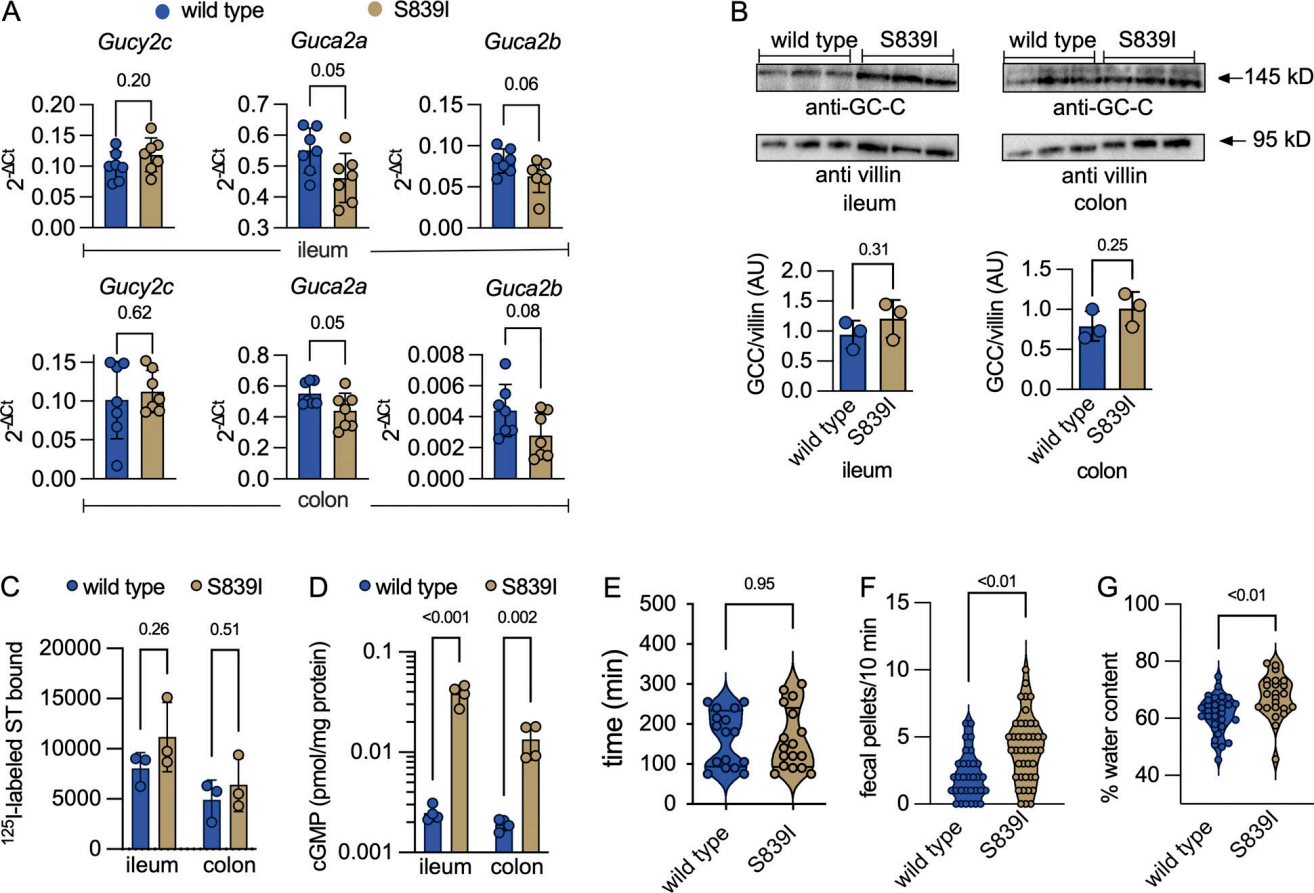
**S839I mice display higher cGMP levels in the small intestine and diarrhea-like symptoms. (A)** Transcript levels of *Gucy2c*,* Guca2a*, and *Guca2b* in the ileum and colon were determined by RTqPCR and normalized to the expression of *Gapdh*. Each dot represents an individual mouse, and values shown are the mean ± SD; P values shown are from unpaired, two-tailed *t* tests with Welch’s correction for each gene. Seven female mice were used for this analysis across experiments repeated twice. **(B)** Western blot analysis of membranes prepared from ileal and colonic epithelial cells was performed using a monoclonal antibody to GC-C, and data shown from three female mice are representative of experiments repeated thrice. The expression of GC-C was normalized to expression of villin. Each dot represents individual mice from the experiment. Values represent mean ± SD, and P values shown are from unpaired, two-tailed *t* tests performed for each tissue with Welch’s correction. The top band in colonic epithelial cells is a nonspecific band since it was seen in membranes prepared from GC-C knockout mice (data not shown). **(C)** Receptor content in IEC membranes prepared from the ileum and the colon. Each dot represents data from membranes prepared from three female mice. Experiments were repeated thrice, and data shown are from one experiment. Values represent the mean ± SD, and P values are from unpaired, two-tailed *t* tests with Welch’s correction. **(D)** Cyclic GMP levels in IECs of wild type and S839I mice. Cyclic GMP levels were estimated using ELISA. Each dot represents an individual mouse, and values represent mean ± SD with P values from unpaired, two-tailed *t* tests with Welch’s correction. Experiments were repeated twice, and data shown are from a single experiment with four female mice. **(E)** Gut transit in wild type and S839I mice, as measured by presence of carmine red in fecal pellets following oral gavage. Each value is from an individual mouse, and the bar represents the mean. Data were analyzed by an unpaired, two-tailed *t* test with Welch’s correction, and P values are shown. For wild type mice, data are shown from nine male and seven female mice. For S839I mice, data are from 12 male and 5 female mice across experiments performed at least thrice. **(F)** Estimation of bowel frequency in wild type and S839I mice. Each value shown represents data from a single mouse, and P values from an unpaired, two-tailed *t* test with Welch’s correction are shown. For wild type mice, data from 17 males and 15 females are shown, and from S839I mice, data from 26 male and 16 female mice are shown across experiments performed at least thrice. **(G)** Fecal water content in wild type and S839I mice. Fecal weight was measured before and after lyophilization. Each dot represents an individual mouse, and P values shown are from an unpaired, two-tailed *t* test with Welch’s correction. For wild type mice, data are from 20 male mice and 16 female mice. For S839I mice, data are from 15 male mice and 19 female mice across experiments performed at least thrice.

**Figure S3.**
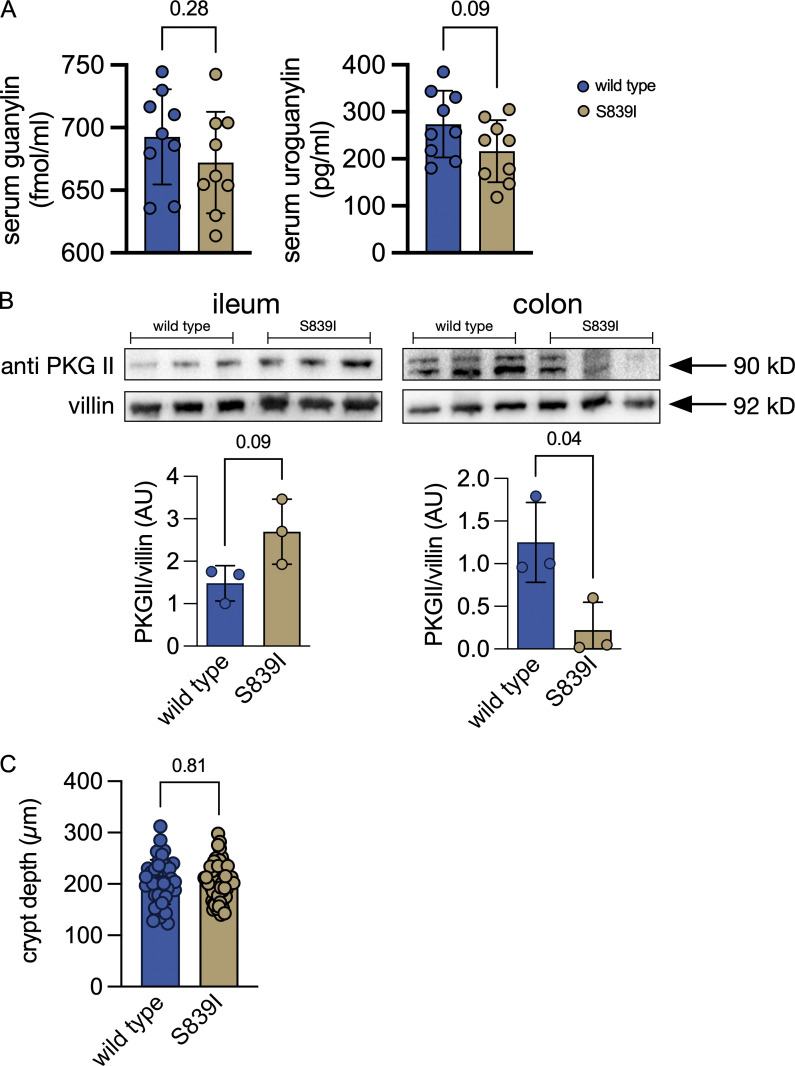
**Circulating levels of endogenous ligands of GC-C, expression of PKGII in enterocytes, and crypt depth in wild type and S839I mice.** **(A)** Levels of endogenous GC-C ligands, guanylin, and uroguanylin were measured in the serum of wild type and S839I mice by ELISA. Four male and five female mice of each genotype were used. The mean ± SD is shown. The P value was obtained from an unpaired, two-tailed *t* test with Welch's correction. **(B)** Western blot showing PKGII expression in the membrane of ileal and colonic enterocytes of wild type and S839I mice. PKGII expression was normalized to villin. Graph shows densitometric analysis, with each dot representing an individual mouse. Three female mice of each genotype were used, and mean ± SD is shown. The P value is obtained from an unpaired, two-tailed *t* test with Welch's correction. **(C)** Colonic sections were imaged using an Axio-Observer Z1 microscope (Carl Zeiss Microimaging). 10–15 distinct, well-defined crypts were identified per section of colonic tissue, and the length of all the crypts was measured using Axiovision software. Data shown are crypt depths measured from four male mice.

Transcript levels of *Gucy2c* and uroguanylin (*Guca2b*) were similar in the ileum and colon of wild type and S839I mice, while levels of guanylin (*Guca2a*) were significantly lower in S839I mice (Fig. 2 A). The gut is the major site of synthesis of guanylin and uroguanylin; therefore, serum levels of the propeptides are reflective of levels produced in the gut. However, we did not see a significant decrease in propeptide hormone levels in S839I mice sera (Fig. S3). Western blotting revealed that GC-C expression was similar in wild type and S839I mice (Fig. 2 B), as was ST binding to membranes prepared from epithelial cells (Fig. 2 C).

The presence of GC-C harboring hyperactivating mutations in patients’ gut was hypothesized to result in elevated intra-epithelial cell cGMP in response to guanylin and/or uroguanylin (Fiskerstrand et al., 2012; Müller et al., 2016). As shown in Fig. 2 D, steady-state levels of cGMP in epithelial cells were ∼5- to 10-fold higher in S839I mice. Therefore, mutant mouse GC-C indeed elicited a greater response in terms of cGMP production in response to the endogenous ligands.

Patients with FGDS demonstrated a delayed gut transit time due to regurgitation of gut contents in the small intestine (von Volkmann et al., 2017, 2016). We estimated gut transit in wild type and S839I mice but observed no change in S839I mice (Fig. 2 E).

Patients with activating mutations in *GUCY2C* pass multiple, watery stools (Fiskerstrand et al., 2012). We monitored the number of fecal pellets passed by mice in 10 min and observed that S839I mice passed a greater number of fecal pellets (Fig. 2 F). The water content in feces produced by S839I mice was higher (Fig. 2 G). These phenotypes mirror the symptoms of diarrhea seen in FGDS, suggesting that S839I mice can be used to understand processes that regulate the frequent episodes of bowel evacuation seen in FGDS patients.

### CFTR activation, Nhe3 inhibition, and increased luminal and fecal sodium in S839I mice

**Figure 3.**
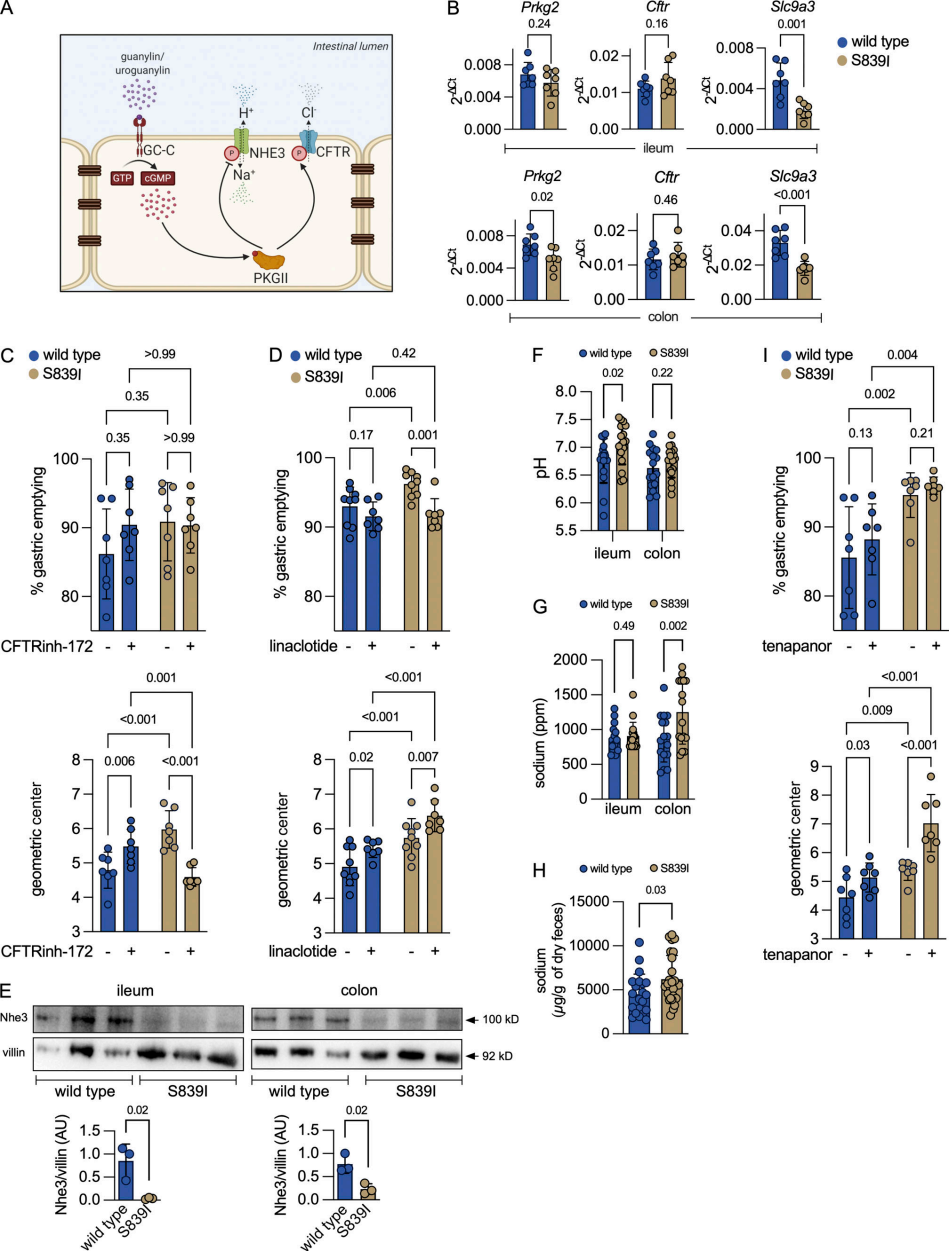
**Elevated cGMP levels activate CFTR and inhibit Nhe3 in vivo. (A)** Signaling events regulated by GC-C in the IEC. Phosphorylation of CFTR by PKGII increases chloride and bicarbonate secretion. Phosphorylation of NHE3 inhibits Na^+^ uptake. Image created using BioRender. **(B)** Transcript levels of genes downstream of GC-C. Each value represents data from an individual mouse, and the mean ± SD is shown. P values obtained from individual unpaired, two-tailed *t* tests with Welch’s correction are shown. Data are from seven female mice across two experiments. **(C)** GE and small intestinal transit after oral gavage with vehicle (TPGS) or CFTR(inh)-172. Values shown are from individual mice, and the mean ± SD is indicated. Two-way ANOVA with a two-stage linear step-up procedure of Benjamini, Krieger, and Yekutieli was performed, and adjusted P values are shown. Data are from experiments performed twice with a total of eight male and eight female mice of each genotype. **(D)** GE and small intestinal transit after oral gavage with buffer or linaclotide. Values shown are from individual mice, and the mean ± SD is indicated. Two-way ANOVA with a two-stage linear step-up procedure of Benjamini, Krieger, and Yekutieli was performed, and adjusted P values are shown. Data are from experiments performed twice with a total of eight male and eight female mice of each genotype. **(E)** Western blot showing Nhe3 expression in the membrane fraction of ileal (left) and colonic (right) epithelial cells. For samples from the ileum, 150 µg of protein was loaded, and for the colon, 80 µg of protein was loaded. The Nhe3 levels were normalized to villin. Graphs shown below depict densitometric analysis and represent the mean ± SD with data analyzed by an unpaired, two-tailed *t* test. Data are representative of three female mice. **(F)** Luminal pH in the ileum and the colon. Values shown are mean ± SD with data from individual mice, and P values shown are from an unpaired, two-tailed *t* test with Welch’s correction. Data are shown from 17 male and 9 female mice of both genotypes across experiments performed thrice. **(G)** Luminal sodium levels in the ileum and the colon. Values shown are mean ± SD with data from individual mice, and P values shown are from an unpaired, two-tailed *t* test with Welch’s correction. Data are from eight male and nine female mice, and experiments were performed twice. **(H)** Sodium content in fecal pellets collected. Values shown are the mean ± SD with data shown from individual mice, and P values shown are from an unpaired, two-tailed *t* test with Welch’s correction. Data from wild type mice are from 9 male and 12 female mice and from 12 male and 13 female S839I mice across experiments performed thrice. **(I)** GE and small intestinal transit after oral gavage with vehicle (TPGS) or the Nhe3 inhibitor (tenapanor). Values shown are from individual mice, and the mean ± SD is indicated. Two-way ANOVA with a two-stage linear step-up procedure of Benjamini, Krieger, and Yekutieli was performed, and adjusted P values are shown. Experiments were performed twice with a total of eight male and six female mice of each genotype.

Elevated cGMP levels in IECs stimulate enhanced chloride and bicarbonate secretion through CFTR following phosphorylation by cGMP-dependent protein kinase G II (Fig. 3 A; Golin-Bisello et al., 2005; Lin et al., 1992). In addition, inhibitory phosphorylation of the sodium-hydrogen exchanger NHE3 (SLC9A3) results in reduced sodium ion import into cells and consequent increase in luminal and fecal sodium (Chen et al., 2015). Transcript levels of *PrkgII* were reduced in the colon of S839I mice as was the level of the protein (Fig. S3 B), suggesting that some effects of cGMP in the colon could be PKGII independent. While *Cftr* transcripts were similar in both wild type and S839I mice, levels of *Nhe3* were lower in both the ileum and colon in S839I mice (Fig. 3 B).

We measured small intestinal transit rates in wild type and S839I mice. Gastric emptying (GE) was slightly enhanced in wild type mice following treatment with the Cftr inhibitor, but not significantly, though GE is reported to be enhanced in CFTR patients (Collins et al., 1997). No change in GE was seen in S839I mice (Fig. 3 C, upper panel) either in the presence or absence of a Cftr inhibitor. However, the extent of migration of the dye in the small intestine, as measured by the geometric center (GC), was significantly higher in S839I mice (Fig. 3 C, lower panel) treated with vehicle alone. On administration of the Cftr inhibitor, an increase in GC in wild type mice was seen (Fig. 3 C, lower panel), which agrees with the paradoxical observation that upper small intestinal transit is increased in cystic fibrosis patients (Hedsund et al., 2012). Importantly, a dramatic reduction in the GC was seen in S839I mice treated with the Cftr inhibitor (Fig. 3 C, lower panel), demonstrating that the increased basal transit rate in S839I mice was almost solely due to CFTR activation.

We then administered linaclotide to more potently activate GC-C (Bryant et al., 2010). GE was higher in S839I mice (Fig. 3 D, upper panel) when mice were gavaged with buffer alone, in contrast to what was seen in mice gavaged with the vehicle used to dissolve the Cftr inhibitor (Fig. 3 C, upper panel). GC-C and uroguanylin expression has been reported in the stomach of mammals, albeit at low levels (Date et al., 1999; London et al., 1997), and the increased GE could be a consequence of hyperactive GC-C in the stomach. On linaclotide treatment, the GC was increased in both wild type and S839I mice (Fig. 3 D, lower panel). However, transit was significantly higher in S839I mice. Therefore, ligand-mediated activation of hyperactive GC-C causes more rapid migration down the small intestine.

We had observed a transcriptional down-regulation of *Nhe3* in both the ileum and the colon of S839I mice in comparison with wild type mice (Fig. 3 B). We monitored expression of Nhe3 and saw a significant reduction in protein expression in both the ileum and colon of S839I mice (Fig. 3 E). Increased bicarbonate efflux from the cell, due to elevated cGMP levels and Cftr activity, along with reduced expression of Nhe3 should increase sodium levels and luminal pH along the gut. In agreement with this, luminal pH in the ileum of S839I mice was higher than in wild type mice (Fig. 3 F). However, a significant increase in luminal sodium was observed only in the colon (Fig. 3 G) and was correlated with higher fecal sodium content (Fig. 3 H). This suggests that Nhe3 may not be the main contributor to sodium import in the small intestine. However, down-regulation of *Nhe3* coupled with inhibition of its activity due to elevated cGMP levels in the colon manifests in enhanced excretion of fecal sodium, as seen in children with hyperactivating mutations in GC-C that show congenital sodium secretory diarrhea (Müller et al., 2016).

We evaluated the extent to which Nhe3 is inhibited by elevated cGMP levels in the gut in S839I mice by administering tenapanor, a specific Nhe3 inhibitor. While GE was again higher in S839I mice administered vehicle alone (Fig. 3 I, upper panel), no further increase was seen in both wild type or S839I mice following administration of tenapanor. However, in agreement with earlier studies (McHugh et al., 2018), administration of the inhibitor to wild type mice increased the GC (Fig. 3 I, lower panel). In S839I mice, the already elevated basal transit was further enhanced by tenapanor treatment (Fig. 3 I). We attribute this enhanced increase in small intestinal transit to the prevalent lower levels of Nhe3 present in S839I mice and efficient inhibition by tenapanor.

### S839I mice are more susceptible to dextran sulfate sodium (DSS)–induced colitis

**Figure 4.**
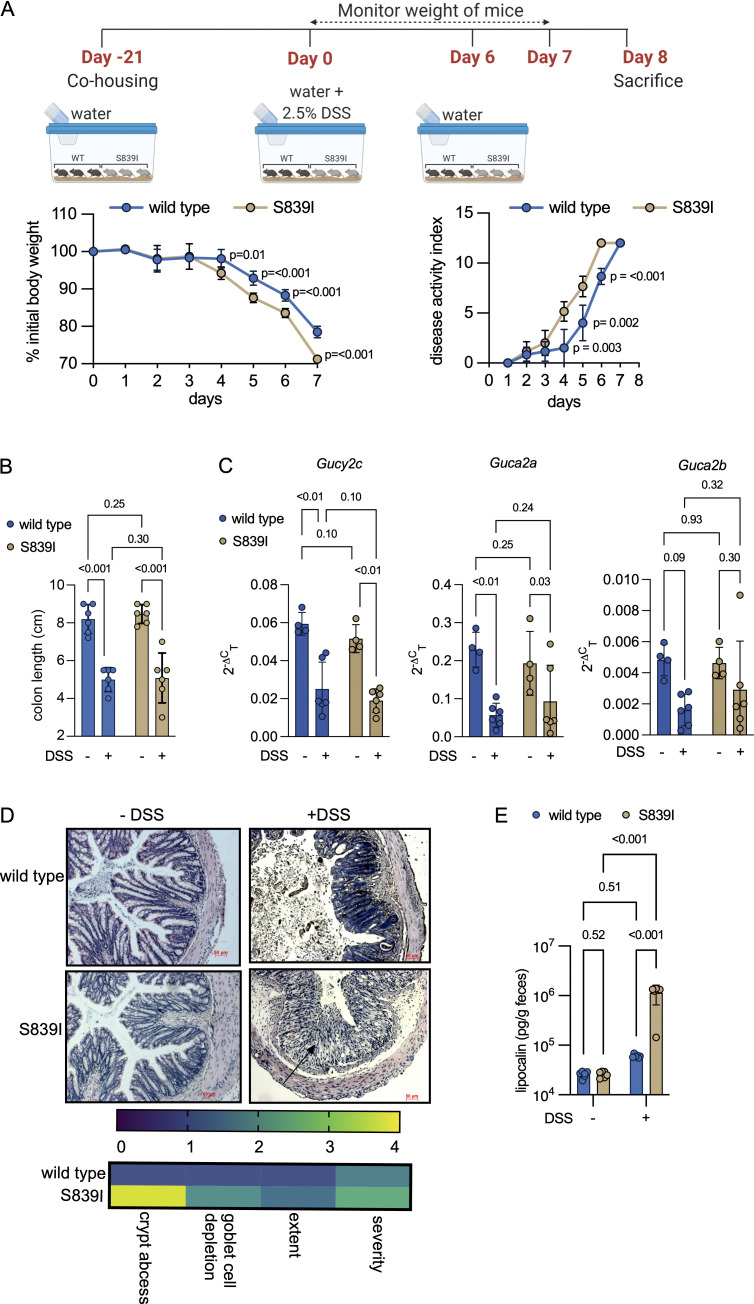
**Mutant S839I mice show enhanced susceptibility to chemical-induced colitis. (A)** Top: Depicts the details of DSS administration and monitoring of mice following induction of colitis. Left: Graph shows the mean weight loss observed following DSS exposure (six mice each). P values shown are from unpaired, two-tailed *t* tests with Welch’s correction at indicated time points. The experiment was repeated thrice, and data shown are from a single experiment and are the mean ± SD. Right: The disease activity index was scored, and P values are from unpaired, two-tailed *t* tests with Welch’s correction at indicated time points from the experiment shown in A. **(B)** Mice were treated with DSS or water and sacrificed on day 7. Each dot represents the colon length of an individual male mouse (six in total) across two experiments. Data shown are the mean ± SD and were analyzed by two-way ANOVA with a two-stage linear step-up procedure of Benjamini, Krieger, and Yekutieli, and adjusted P values are shown. **(C)** RTqPCR analysis of *Gucy2c* and its ligands following DSS treatment. Values shown are from individual male mice from a single experiment. The mean ± SD is shown. Data were analyzed by two-way ANOVA with a two-stage linear step-up procedure of Benjamini, Krieger, and Yekutieli, and adjusted P values are shown. Experiments were repeated thrice. **(D)** Sections were prepared from the distal colon of untreated or DSS-treated mice. Sections shown are representative of data from four mice, and the arrow indicates significant infiltration of immune cells into the colonic tissue in S839I mice. Scale bars, 50 µm. Images were evaluated for histomorphological changes. The mean across four wild type and four S839I mice is shown in the heat map. **(E)** Fresh feces were collected from each mouse before and 4 d after DSS treatment. Fecal lipocalin levels were estimated using ELISA. Values shown are the mean ± SD from six individual male mice. Data shown are the mean ± SD and were analyzed by two-way ANOVA with a two-stage linear step-up procedure of Benjamini, Krieger, and Yekutieli, and adjusted P values are shown. Experiments were repeated twice.

Patients harboring activating mutations in *GUCY2C* present with varying degrees of inflammation in the gut (such as esophagitis, irritable bowel syndrome [IBS], CD, and ulcerative colitis [UC]; Fiskerstrand et al., 2012; Müller et al., 2016). Administration of DSS to mice causes death of epithelial cells and compromises barrier function (Wirtz et al., 2017). We administered DSS to mice and monitored weight loss and damage to the colon. S839I mice were more susceptible to DSS as evidenced by greater weight loss (Fig. 4 A, left panel) and higher disease activity index (Fig. 4 A, right panel). Colonic shortening was observed in both wild type and S839I mice after DSS treatment (Fig. 4 B).

Transcript levels of GC-C and guanylin are reduced in biopsies taken from human UC and CD patients (Brenna et al., 2015; Lan et al., 2016). Following the induction of colitis by DSS, transcript levels of *Gucy2c* and *Guca2a* were reduced in both wild type and S839I mice (Fig. 4 C).

Histological evaluation of the colon in wild type and S839I mice revealed no difference in colon architecture or changes in crypt depth, indicating that IEC turnover was similar in S839I mice (Fig. S3 C). After DSS treatment, a greater degree of crypt abscesses and destruction of colonic mucosa was observed in S839I mice (Fig. 4 D). Concomitant with greater mucosal damage, fecal lipocalin, a sensitive marker for inflammation in the gut (Chassaing et al., 2012), was increased in S839I mice (Fig. 4 E). Taken together, our results show that S839I mice harboring an activating mutation in *Gucy2c* reveal roles of cGMP in regulating gut function and enhanced colonic susceptibility to damage in a colitis model. Notably, GC-C knockout mice are resistant to DSS-induced colitis (Steinbrecher et al., 2011).

To explore global changes seen in the gut because of the presence of hyperactive GC-C, we took unbiased approaches by characterizing the fecal microbiome and the transcriptome in colonic tissue.

### Dysbiosis in S839I mice is correlated with changes seen in IBD and colitis

**Figure 5.**
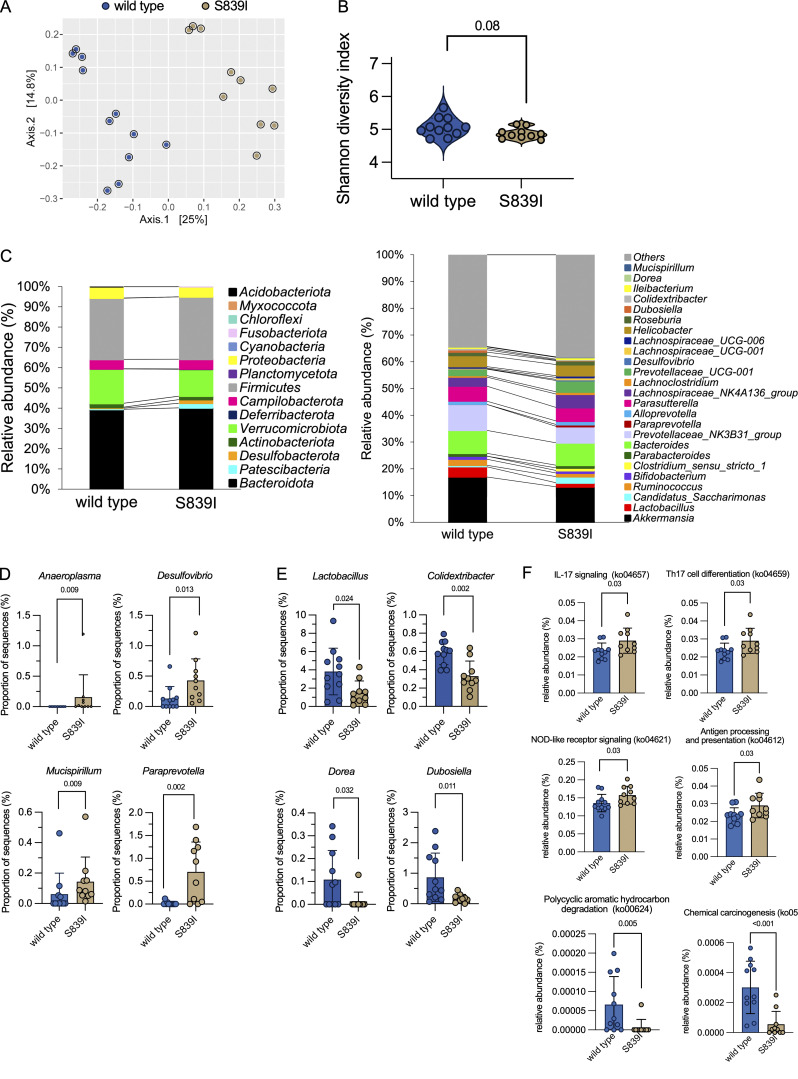
**Mutant S839I mice display gut microbial dysbiosis. (A)** Fecal DNA was processed to obtain 16S rRNA abundance (eight female and three male wild type mice; seven female and three male S839I mice). **(B)** Comparison of α diversity measured using Shannon diversity index. Statistical significance was determined using Wilcoxon rank sum test (P < 0.05). **(C)** Stacked-bar plot representing the phylum-level (left panel) and genus-level (right panel) relative abundance of bacterial communities in the fecal microbiome of wild type and S839I mice. **(D** **and** **E)** Genera that were significantly increased (D) or decreased (E) in the fecal microbiome of S839I mice. Data shown in are mean ± SD. Comparisons were done using Kruskal–Wallis test, and those with P < 0.05 are shown. **(F)** Predicted functional composition of the fecal microbiome. KEGG orthology function within the KEGG pathways for IL-17 signaling (ko04657), Th17 cell differentiation (ko04659), and NOD-like receptor signaling (ko04621). Data shown are mean ± SD. Statistical analysis was conducted using a Kruskal–Wallis test with Tukey-Kramer post hoc test.

The altered pH and sodium ion concentrations in the gut lumen may result in dysbiosis that could predispose to colitis. We therefore performed 16S ribosomal RNA (rRNA) gene amplicon sequencing of fecal samples collected from wild type and S839I mice. Unconstrained ordination through principal component analysis with Bray-Curtis dissimilarity metrics displayed a clear separation between the two sets of mice (analysis of similarities P value = 0.001; Fig. 5 A). There was a reproducible trend of reduced α diversity (P < 0.1) in the fecal microbiome of S839I mice (Fig. 5 B), and relative abundance plots demonstrated differences at the phylum- and genus-levels (Fig. 5 C).

An increased abundance of potential opportunistic pathogens (*Anaeroplasma*, *Desulfovibrio*, *Mucispirillum*, and *Paraprevotella*) was seen in S839I (Fig. 5 D). Members of the genus *Paraprevotella* are associated with colonic CD and produce succinic acid, increased levels of which are reported to be associated with microbiome dysbiosis and intestinal inflammation in patients with IBD and animal models of chronic colitis (Macias-Ceja et al., 2019; Walters et al., 2014). The genus *Mucispirillum* is also significantly higher in S839I mice (Fig. 5 D). Exposure of *Nod2*^−/−^*Cybb*^−/−^ C57BL/6 mice to a mucus-dwelling Gram-negative pathobiont of rodents, *Mucispirillum schaedleri,* has been reported to trigger the development of CD-like colitis (Caruso et al., 2019). *Desulfovibrio* was significantly enriched in S839I mice as seen in the colonic mucosal and fecal microbiome of UC and CD patients (Rowan et al., 2010).

Levels of protective bacteria such as *Colidextribacter*, *Dorea* (short-chain fatty acid producers), *Dubosiella*, and *Lactobacillus* (possessing anti-inflammatory properties) were significantly reduced in S839I mice (Fig. 5 E). *Colidextribacter* and *Dorea* belong to *Clostridiales* cluster IV and *Clostridium* cluster XIVa in the phylum Firmicutes, respectively. These taxa are known to produce short-chain fatty acid and are reported to be less abundant in the ileal biopsy and fecal samples isolated from CD patients (Nagao-Kitamoto and Kamada, 2017). *Lactobacillus* strains restore the commensals and gut homeostasis in intestinal disorders (Blaser, 2014). Therefore, the lower abundance of these genera in S839I mice suggests that these animals may be more susceptible to environment-induced colitis and inflammation in the gut, as reported in FGDS patients (Fiskerstrand et al., 2012).

Analysis of predicted functional consequences of the variation in abundance of taxa between the mice (Narayan et al., 2020) indicated significant differences in 28 KEGG pathways (Table S3). Those linked to host immunity were enriched in S839I mice and included NOD-like receptor signaling, antigen processing and presentation, IL17 signaling, and Th17 cell differentiation (Fig. 5 F). KEGG pathways for bacterial chemotaxis and flagellar assembly, both associated with cell motility in the microbiome, were significantly higher in S839I mice (Table S3). Pathways that were decreased were linked to polycyclic aromatic hydrocarbon degradation, suggesting that S839I mice may have higher levels of these genotoxic compounds in their gut, which could predispose them to carcinogenesis; in contrast, pathways linked to chemical carcinogenesis were reduced (Fig. 5 F).

In summary, significant differences in the microbiome of S839I mice resemble changes seen in IBD and FGDS patients (Tronstad et al., 2017) and more recent data related to the fecal microbiota seen in microscopic colitis patients (Hertz et al., 2021). Therefore, the underlying disturbances in colonic epithelial function and/or an imbalance in fluid and ion secretion due to the activating *Gucy2c* mutation has profound effects on the gut flora.

### RNA sequencing (RNA-seq) analysis reveals an inflammatory gene signature in the gastrointestinal tract

**Figure 6.**
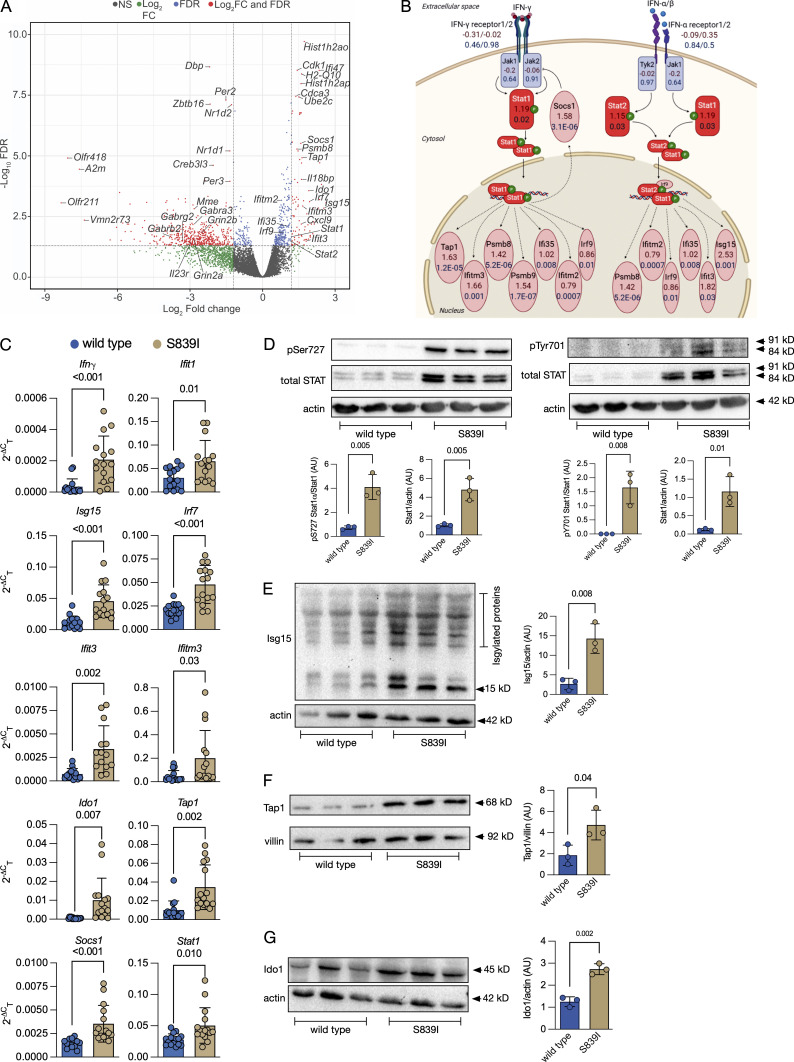
**RNA-seq analysis in the distal colon of wild type and mutant S839I mice. (A)** A volcano plot showing differentially expressed protein coding genes in S839I mice. Female mice (four of each genotype) were used for the experiment. **(B)** Transcript levels of differentially regulated genes associated with IFN signaling as analyzed by IPA. Values shown below symbols represent the Log_2_FC seen in the dataset (red) and the FDR (blue). Analysis was performed after a log_2_FC cutoff of −2 and +1.5 and FDR cutoff < 0.05. **(C)** RTqPCR of genes associated with IFN and Stat1 signaling. Each dot represents RNA prepared from individual mice. Data were analyzed by an unpaired, two-tailed *t* test with Welch’s correction. The mean ± SD and P values are shown. Data are shown from seven female and eight male mice of both genotypes. **(D)** Western blot of STAT1 phosphorylated on Ser727 (left panel) or Tyr701 (right panel) in the cytosolic fraction of colonic tissue extracts of wild type and S839I mice. Phosphorylated STAT1 was normalized to total STAT1. Total STAT1 levels were normalized to actin levels. Graphs shown below are densitometric analyses of individual bands and represent the mean ± SD with data analyzed by an unpaired, two-tailed *t* test with Welch’s correction. Each dot represents an individual male mouse. **(E)** Western blot of Isg15 and Isgylated proteins in the cytosolic fraction of colonic tissue extracts. Free Isg15 levels, migrating at 15 kD, were normalized to actin. Graph shown to the right is densitometric analysis of free Isg15 and represents the mean ± SD with data analyzed by an unpaired, two-tailed *t* test with Welch’s correction. Each dot represents an individual male mouse. **(F)** Western blot of Tap1 in the membrane fraction of colonic tissue extracts. Tap1 levels were normalized to villin. Graph shown to the right is densitometric analysis of Tap1 and represents the mean ± SD with data analyzed by an unpaired, two-tailed *t* test with Welch’s correction. Each dot represents an individual male mouse. **(G)** Western blot of Ido1 in the cytosolic fraction of colonic tissue extracts. Ido1 levels were normalized to villin. Graph shown to the right is densitometric analysis of Ido1 and represents the mean ± SD with data analyzed by an unpaired, two-tailed *t* test with Welch’s correction. Each dot represents an individual male mouse.

We compared global gene expression changes in the colon of wild type and S839I mice by RNA-seq analysis, hypothesizing that the pattern of gene expression may explain the susceptibility to inflammation seen in patients. We identified several differentially regulated genes, with a majority being down-regulated (Fig. 6 A). Differentially expressed transcripts with adjusted false discovery rate (FDR) < 0.05 and a log_2_ fold change (FC) of less than −2 and >1.5 yielded 645 down-regulated and 101 up-regulated genes (Fig. 6 A). Ingenuity Pathway Analysis (IPA) identified canonical pathways that are perturbed in S839I mice. Strikingly, increased levels of a large number of genes regulated by IFN signaling (Barrat et al., 2019), called IFN-stimulated genes (ISGs), were observed and included *Ifit1*, *Ifit3*, *Ifitm3*, *Tap1*, *Irf7*, *Isg15*, *Ido1*, and *Socs1* (Fig. 6 B). We validated the expression levels of these genes by RTqPCRand observed that the increase in transcript levels experimentally observed was in concert with that seen in the RNA-seq analysis (Fig. 6 C).

We then looked for evidence of altered regulation of type I, type II, and type III IFN genes that would drive expression of ISGs. No members of the IFN1 and IFNIII families were detected in the RNA-seq, and transcripts were also not identified by RTPCR (data not shown). *Ifng*, however, could be detected by RTPCR (Fig. 6 C), and levels were higher in S839I mice.

Many of the ISGs are STAT1 targets, including *Socs1*, which is a negative regulator of cytokine signaling. *Stat1* was up-regulated in S839I mice as was *Socs1*, further validating the results seen in RNA-seq analysis (Fig. 6 C). We then looked for expression of Stat1 and its phosphorylated forms by Western blotting using extracts prepared from whole colonic tissue. A significant increase in total levels of Stat1, along with phosphorylated Stat1 (at Ser727 and Tyr701), was observed in S839I mice. An increase in total Stat1 was also seen, possibly since Stat1 autoregulates its own transcription (Fig. 6 D). The significant increase in total STAT1 could also perhaps be a consequence of direct transcriptional induction in response to elevated cGMP levels. Further, levels of Isg15 (Fig. 6 E), Tap1 (Fig. 6 F), and Ido1 proteins (Fig. 6 G), which are all regulated by Stat1 and whose transcripts were increased in S839I mice (Fig. 6 C), were increased. Notably, increased STAT1 activity is associated with severity of disease in IBD in patients (Cordes et al., 2020; Schreiber et al., 2002).

Given the increase in total Stat1 seen in S839I mice, it is possible that a fraction may remain unphosphorylated. Induction of ISGs mediated by unphosphorylated STAT1, as part of a tripartite transcription complex of STAT1, STAT2, and IRF9, was reported earlier in colonic cell lines (Wang et al., 2017). Indeed, RNA-seq analysis indicated that *Stat2* and *Irf9* were also up-regulated in S839I mice. Expression of ISGs by unphosphorylated STAT1 is proposed to be a priming mechanism to overcome microbial infection (Cheon et al., 2013).

**Figure 7.**
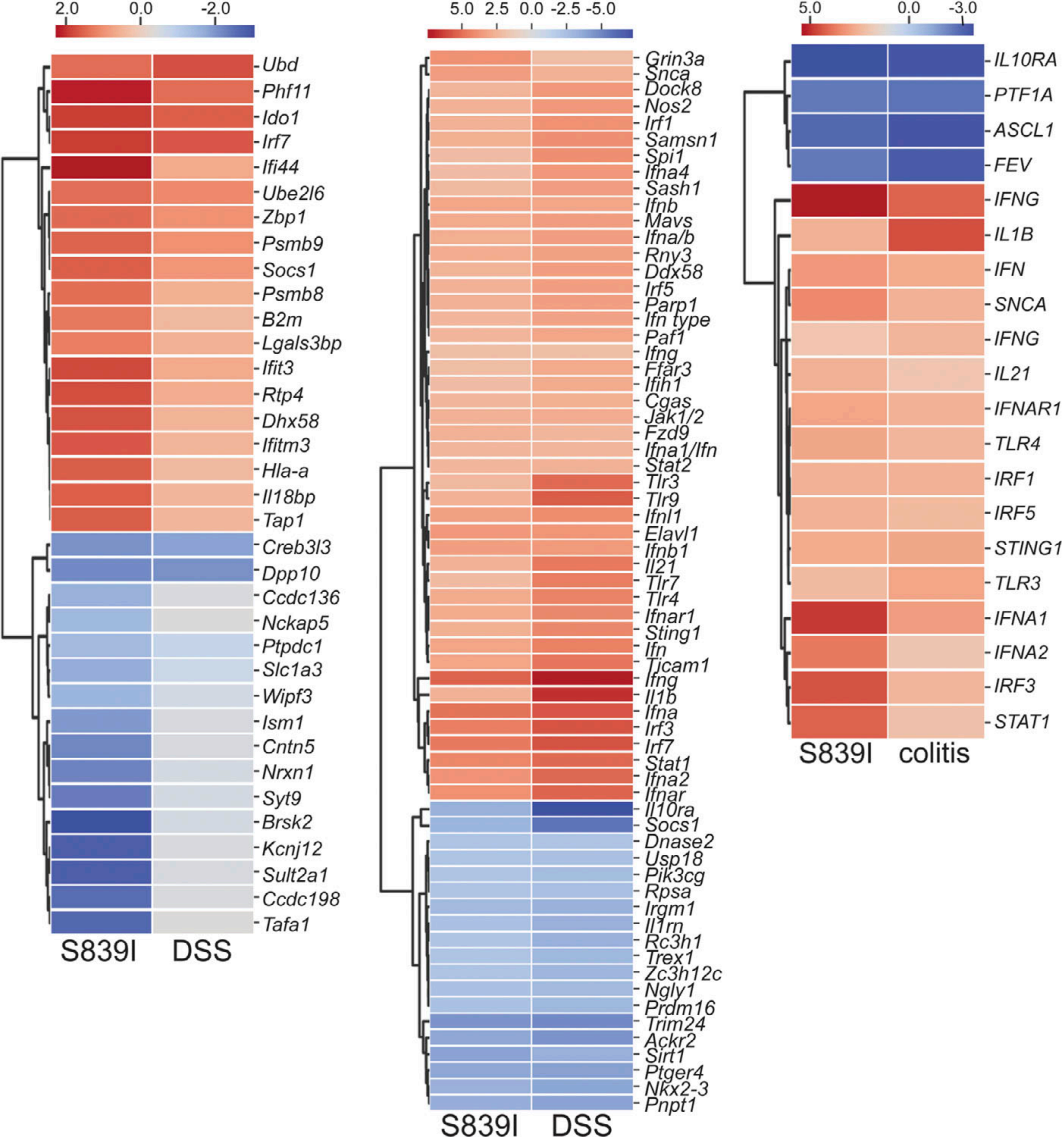
**Comparison of colonic transcriptomes of S839I mice, murine colitis models, and human UC patients.** Left panel shows genes similarly regulated in DSS-treated mice and S839I mice. The center panel shows predicted upstream regulators in S839I mice and DSS-treated mice as identified by IPA. Right panel shows upstream regulators identifiable by IPA in S839I mice and in colonic biopsies from UC patients. Upstream regulators with z-score cutoff of ≤2 or >2 are shown.

The enhanced expression of ISGs in S839I mice may be a cause of the baseline pathology and consequent susceptibility to severe colitis we report here (Fig. 4). We therefore used the Analysis Match function in IPA to compare altered gene expression seen in S839I mice with that of mice following DSS treatment. A significant number of genes showed the same trend of regulation in S839I mice and mice treated with DSS (Fig. 7), indicating that the inflammatory signatures following DSS treatment appeared to be already altered in S839I mice. IPA predicts a z-score of activation or inhibition of gene expression controlled by upstream regulators. Here again, common upstream regulators with comparable activation z-scores were found in S839I mice and those with colitis induced by DSS (Fig. 7).

Finally, upstream regulators in our dataset showed similar predicted z-scores to those seen in colonic biopsies from colitis patients (Zhao et al., 2015; Fig. 7), validating that these S839I mice can indeed serve as a preclinical model to study gut inflammation in humans mediated by hyperactive *GUCY2C* mutations (Crowley et al., 2020).

## Discussion

Here, we describe a novel preclinical model to understand the underlying biological mechanisms seen in patients with rare mutations in *GUCY2C*. Our results show that hyperactivation of GC-C results in loss of overall homeostasis, fluid-ion imbalance, gut microbiota dysbiosis, and susceptibility to colitis. Since there is growing evidence that interorgan communication is the basis for the overall phenotype observed in organisms and FGDS patients harbored the activating mutation in all tissues where GC-C is expressed, we chose to develop a whole-body knockin model.

The increased level of cGMP in IECs in S839I mice (Fig. 2 D) results in an increase in small intestinal transit via activation of Cftr (Fig. 3, C and D). Inhibition of Nhe3 by tenapanor also resulted in enhanced transit (Fig. 3 I). A significant decrease in transcript as well as protein levels of Nhe3 was seen in S839I mice (Fig. 3, B and E). Since lower Nhe3 expression would reduce Na^+^ uptake by the epithelial cell, an increase in luminal sodium in the colon and the feces was seen, but not in the ileum (Fig. 3, G and H). *Nhe3^−/−^* mice displayed diarrhea with impaired fluid absorption, higher luminal sodium ion content in the intestine, and alkalization of the intestinal lumen (Schultheis et al., 1998; Xue et al., 2020), as we see here (Fig. 3 F). While Nhe3 has been reported to regulate sodium levels in mouse ileal tissue (Murtazina et al., 2011), more recent observations indicate that Nhe3 plays a modest role in sodium fluxes in the distal ileum (Stephens et al., 2021). Therefore, there could be additional mechanisms by which sodium levels are maintained in the ileum.

Inactivating mutations in *NHE3* result in congenital sodium diarrhea due to malabsorption of sodium (Janecke et al., 2015). Altered and defective Na^+^ absorption is considered one of the most important factors for diarrhea in patients with IBD (Seidler et al., 2006). A decrease in expression or activity of NHE3 has been documented in mucosal biopsies from patients with IBD (Siddique et al., 2009; Yeruva et al., 2010). Similarly, *Il10^−/−^* mice that develop spontaneous colitis show reduced Nhe3 activity in the enterocyte (Larmonier et al., 2011; Sellon et al., 1998). Therefore, reduced NHE3 activity in the gut of patients with hyperactive *GUCY2C* mutations could contribute to the incidence of Crohn’s-like symptoms and colitis. We recently speculated that impaired intestinal sodium transport and its effects on the microbiome could serve as a major upstream mediator of downstream pathophysiology (Prasad and Visweswariah, 2021).

The reduced transcript levels and protein expression of Nhe3 in S839I mice suggests a role for cGMP (and perhaps PKGII) in reducing *Nhe3* transcription. *Nhe3* transcription is positively regulated by Sp1/Egr-1 transcription factors (Malakooti et al., 2006). Parathyroid hormone–induced inhibition of *Nhe3* transcription in opossum kidney proximal tubule cells was mediated by enhanced Egr-1 binding to the core *Nhe3* promoter and activation of JAK-STAT3 activity (Neri et al., 2015). A recent study indicated that p38 activation in human colonic Caco2 cells was correlated with a reduction in *Nhe3* transcription (Enns et al., 2020). We showed earlier that PKGII activates p38, which in turn results in the increased recruitment of Sp1 to the p21 promoter, resulting in enhanced *p21* transcription (Basu et al., 2014). What remains to be explored in view of our findings described here is whether inhibition of *Nhe3* transcription is a result of crosstalk between cGMP, PKGII, p38, Sp1/Egr-1, and STAT1 in IECs.

Activation of CFTR resulting in increased chloride efflux is the major cause of diarrhea during enteric infections, causing increased chloride and bicarbonate secretion into the lumen of the intestine. Mice with hyperactivation of Cftr display signs of diarrhea and alkalization of intestinal lumen due to increased bicarbonate secretion (Gelfond et al., 2017). We show here that S839I mice also display alkalization of the small intestinal lumen (Fig. 3 F). The increased transit in the small intestine of S839I mice (Fig. 3 C) because of activation of Cftr and inhibition of Nhe3 suggests that CFTR may be targetable in FGDS patients. Inactivating mutations in CFTR lead to meconium ileus in the newborn (Sathe and Houwen, 2017) and intestinal obstructions in adults due to a reduction in gut motility and hydration. This mimics what is seen in patients with inactivating mutations in *GUCY2C* (Bose et al., 2020; Romi et al., 2012; Smith et al., 2015; Woods et al., 2019), indicating the critical role Cftr plays downstream of GC-C signaling.

FGDS patients displayed prolonged gut transit time and small intestinal dysmotility (von Volkmann et al., 2017). There are conflicting results for intestinal transit rate in patients with IBS-C (IBS with constipation) and IBS-D (IBS with diarrhea) in the literature (Ringel-Kulka et al., 2015; Roland et al., 2015), suggesting a complex regulation of gut motility by the enteric nervous system.

S839I mice displayed higher frequency of bowel movements and fecal water content, suggesting diarrhea-like symptoms (Fig. 2, F and G). However, severe watery diarrhea marked by liquid stool in cage bedding and wet and discolored anogenital areas were not observed in S839I mice. This may be partly due to the genetic background of the mice. For example, C57BL/6N mice develop only modest diarrhea upon infection with the murine pathogen *Citrobacter rodentium* (Bhinder et al., 2013), whereas FVB/N mice showed severe diarrhea and mortality (Borenshtein et al., 2008).

Commensal microbiome load and diversity are highly influenced by epithelial ion transport in the intestine (De Lisle, 2007; Gurney et al., 2017; Keely et al., 2012). Loss of GC-C in mice results in gut microbiota dysbiosis with a lower abundance of *Lactobacillus* (Majumdar et al., 2018). Commensal *Lactobacillus* is known to play protective roles against inflammatory diseases by influencing T regulatory cell (T reg cell) functioning. An increased abundance of Proteobacteria with concomitant decrease in *Lactobacillus* is found in patients with IBD (Sartor, 2008). FGDS patients displayed increased abundance of Enterobacteriaceae (γ Proteobacteria), which is associated with intestinal inflammation and symptoms similar to CD (Tronstad et al., 2017). Under physiological conditions, colonic epithelia undergo β-oxidation and deplete luminal oxygen, resulting in an anaerobic environment. However, during inflammation, the colonic epithelial cells lose their capacity of β-oxidation, resulting in increased luminal oxygen, microbiome dysbiosis, and Proteobacteria bloom (Hughes et al., 2017). This increase in Proteobacteria and decrease in *Lactobacillus* are also observed in *Nhe3^−/−^* knockout mice (Larmonier et al., 2013). Thus, the microbial dysbiosis seen in S839I mice resembles that of patients with IBD and mouse models of colitis (Fig. 5).

The beneficial role of GC-C signaling during IBD is implicated from the observation that human patients with IBD display a significant decrease in transcript levels of *GUCA2A*, *GUCA2B*, and *GUCY2C*, and loss of these proteins is linked with the severity of the disease in patients (Brenna et al., 2015; Lan et al., 2016). We saw a similar change in our mice following DSS treatment (Fig. 4 C). However, paradoxically, patients with the p.S840I mutation in GC-C display signs of CD, IBS, and obstruction in the ileum due to inflammation (Fiskerstrand et al., 2012). Perhaps the decreases in GC-C and ligand expression following DSS treatment are compensatory mechanisms that could counteract increased cGMP levels seen in S839I mice. In addition, pathways not directly regulated by cGMP could also be misregulated in these mice, which, in turn, could feed back into the regulation of GC-C and its ligands. Since mice lacking *Nhe3* display a susceptibility to DSS-induced colitis (Kiela et al., 2009), electrolyte flux and imbalance in the intestine, coupled with alterations in the microbiome, may be the distal drivers of increased susceptibility to chemical-induced colitis (Prasad and Visweswariah, 2021).

RNA-seq revealed that several genes linked to gut inflammation and colitis are misregulated in S839I mice. Indeed, the pattern of gene expression is like that seen in the colon of mice treated with DSS (Fig. 7) and, importantly, IBD patients (Lamas et al., 2016; Zhao et al., 2015). Almost all the genes shown to be up-regulated in biopsies from active UC patients (Zhao et al., 2015) are similarly up-regulated in S839I mice. These include *STAT1*, *IRF1*, *IRF9*, *IFIT2*, *IFIT3*, *IFITM2*, *OAS2*, and *ISG15*. We show here that a significant increase in total and phosphorylated Stat1 levels are seen in S839I mice (Fig. 6, C and D), which would contribute to the increased expression of ISGs. Due to the significant increase in total Stat1 in S839I mice, there may be a significant fraction of unphosphorylated Stat1 in the tissue that could also contribute to ISG expression. This noncanonical STAT signaling via unphosphorylated STATs or by Ser727 monophosphorylated STATs was described earlier on viral infection (Cheon et al., 2013). For example, phosphorylated STATs are dephosphorylated after entering the nucleus, but expression of target genes continues (Bandyopadhyay et al., 2008; Cheon and Stark, 2009). Therefore, constitutive expression of hyperactive S839I in the gut of these mice results in a chronic state of STAT1 activation. We therefore suggest that GC-C via cGMP modulates STAT1 content and activity in the intestinal epithelium, leading to a basal inflammatory signal observed in S839I mice that is exacerbated in the presence of a colitis-inducing agent. In agreement with this is the fact that inflammation was reduced in *Stat1*^−/−^ mice following DSS treatment (Bandyopadhyay et al., 2008), and an *Ido1*^−^*^/^*^−^ mouse (*Ido1* is up-regulated in S839I mice; Fig. 6, C and G) shows a reduced severity to DSS-induced colitis (Shon et al., 2015).

In summary, we have developed a mouse model for FGDS and have identified GC-C as a key regulator of intestinal homeostasis and healthy gut functioning. Our results show the myriad consequences of hyperactivation of GC-C in the intestinal epithelium, including diarrhea, gut microbiota dysbiosis, and susceptibility to intestinal inflammation. This mouse model opens opportunities to study the role of cGMP in the gut and aid in the identification of various targets to inhibit hyperactive GC-C signaling using ex vivo organoid cultures. Importantly, this mutant mouse may serve as a model for ST-mediated diarrhea due to Enterotoxigenic *E. coli* infection, which is a cause of mortality and morbidity in children in developing countries.

## Materials and methods

### Biochemical characterization of S839I

Mouse GC-C cDNA was isolated from mouse intestine, cloned in pBKS, and used as a template for generation of the S839I mutation using site-directed mutagenesis (Shenoy and Visweswariah, 2003). Heterologous stable expression of mouse GC-C was achieved in HEK293E cells using retroviral transduction. Briefly, retrovirus-like particles were packaged in HEK293E cells using the pMX plasmid vector containing either wild type or mutant mouse GC-C, pCMV-Gag-Pol, and pCMV-VSV-G (kind gifts from Dr. Avinash R. Shenoy, Imperial College London, London, UK). Following transduction, transduced cells were grown and maintained in medium containing 2 µg/ml of puromycin to generate cell lines stably expressing wild type or mutant GC-C.

Membrane fractions from stably expressing cell lines were prepared as described earlier (Saha et al., 2009), and GC-C expression was confirmed by Western blot analysis using a monoclonal antibody (4B11) raised to the kinase homology domain of GC-C available in the laboratory. Densitometric analysis was performed using Bio-Rad Image Lab software. Membranes were used for competitive binding assays and Ki calculated as described earlier (Cheng and Prusoff, 1973). In vitro guanylyl cyclase assays in the presence of MnGTP as substrate were performed as detailed earlier (Saha et al., 2009).

Ligand-stimulated GC-C activity in intact cells was measured by addition of indicated concentrations of ST or mouse uroguanylin (Bachem) for 30 min. Cyclic GMP produced was quantitated using radioimmunoassay (Saha et al., 2009). Cyclic GMP produced by cells was normalized to the receptor amounts present on the surface of the cell, which was determined using ^125^I-labeled ST_Y72F_. Receptor content either on the surface of cells or in membrane preparations was estimated using ^125^I-labeled ST_Y72F_ peptide, and the Bmax (that is, the concentration of receptor present in the sample) was calculated using the equations below.

Bmax=[specific·bound]/fractional·occupancy]=[specific·bound]/[radioligand]/(Ki+[radioligand]),

where Bmax is the total number of receptors expressed; [specific bound] is the concentration of specific bound ^125^I-labeled ST_Y72F_; [radioligand] is the concentration of ^125^I-labeled ST_Y72F_ used in the assay, which is negligible compared with the experimentally determined Ki; and Ki is the apparent dissociation equilibrium constant, which is obtained from the IC_50_ (half-maximal inhibitory concentration) data. For ST peptide, this is taken as 6.7 nM.

### Generation of *Gucy2c^S839I/S839I^* mice

*Gucy2c^S839I/S839I^* (S839I) mice were generated via FLP-FRT recombination by Taconic Denmark (Fig. S1 A). Briefly, a p.S839I mutation was introduced in exon 22, and a puromycin cassette flanked with FRT sites was introduced in intron 21. Targeting vectors were generated using BAC clones from the C57BL/6J RPCIB-731 BAC library and were transfected into the Taconic Artemis C57BL/6N Tac ES (embryonic stem) cell line. Homologous recombinant clones were selected using positive (PuroR) and negative (Thymidine kinase) selection. Following generation of clones, superovulated BALB/c females were mated with BALB/c males. Blastocysts were isolated from the uterus at 3.5 d after coitum. The blastocysts were microinjected with C57BL/6NTac ES cells with p.S839I mutation in *Gucy2c*. After recovery, eight injected blastocysts were transferred to each uterine horn of 2.5 d after coitum, pseudopregnant NMRI females. Chimerism was measured by coat color contribution of ES cells to the BALB/c host (black/white). Chimeric mice were bred to C57BL/6N Tac females. C57BL/6N Tac female mating partners were mutant for the presence of a recombinase gene (Flp-Deleter). Germline transmission of the point mutation was identified by the presence of black C57BL/6N Tac offspring.

Two breeding pairs of homozygous mice were received and backcrossed with C57BL/6N Tac wild type mice for 10 generations. All procedures were performed in agreement with the Control and Supervision Rules, 1998 of the Ministry of Environment and Forest Act (Government of India) and the Animal Ethics Committee of the Indian Institute of Science (Approval CAF/Ethics/547/2017). All animals were bred and housed in the same vivarium. Mice were housed in a clean air facility in multiple cages and separated on the basis of sex and genotype. The temperature was maintained at 22 ± 2°C, humidity was maintained at 55% ± 10%, and the mice were maintained on a 12-h light/dark cycle. Mice had access to laboratory chow and water ad libitum. Chow was procured from Altromin International and contained ∼24% protein, 6% oil, and 3% dietary fibers. Mice of both sexes were used for experiments unless specified.

Genomic DNA for genotyping was isolated by the HotShot method (Truett et al., 2000). Briefly, 2 mm of a tail snip was incubated in 75 µl of lysis buffer (25 mM NaOH and 0.2 mM EDTA) at 95°C for 1 h. The tube was then cooled to room temperature, and 75 µl of neutralization buffer (40 mM Tris HCl, pH 5.5) was added. Following centrifugation at 3,000 rcf for 5 min, an aliquot of the supernatant was taken for PCR. A PCR using 2 µl of the supernatant was performed with primer sets Gucy2c_27 (5′-TGA​ACA​GTA​CCC​AGG​AGA​TTA​GG-3′) and Gucy2c_28 (5′-AAC​AGT​TGC​AGA​ATC​CTT​GAG​G-3′) as indicated in Fig. S1 B. The *Gucy2c^S839I/S839I^* allele gave a 371-bp product, while the *Gucy2c^WT/WT^* allele gave a 302-bp product (Fig. S1).

For all experiments, we used the Experimental Design Assistant (RRID:SCR_017019; https://eda.nc3rs.org.uk) to calculate the number of animals required for the experiments. Experimental Design Assistant considers the 3Rs (Replacement, Refinement, and Reduction) in its analysis and estimation of animal numbers needed for an experiment.

### Metabolic cage study

Mice (7–8 wk old) were weighed and housed individually in cleaned and autoclaved metabolic cages (Techniplast). Mice were provided with weighed amounts of feed and measured amounts of water. The temperature of the room, the amount of feed and water intake, and fecal and urine output were recorded every day for 6 d consecutively. The weight of the mice after 6 d of housing in the metabolic cage was recorded, and mice were returned to their original cages.

### RNA isolation and reverse transcription quantitative real-time PCR (RTqPCR) analysis

Mice were sacrificed by CO_2_ asphyxiation, and ∼1-cm piece of the terminal ileum and distal colon was collected and snap-frozen in TRI Reagent (RNAiso Plus; TaKaRa). The RNA was isolated using the Qiagen RNeasy mini kit, according to the manufacturer’s protocol, and 4 µg of RNA was reverse transcribed to cDNA using RevertAid reverse transcription (Thermo Fisher Scientific). Real-time PCR was performed using SYBR Premix Ex Taq (Tli RNase H Plus) on a CFX96 Touch real-time PCR detection system (Bio-Rad). The housekeeping gene glyceraldehyde 3-phosphate dehydrogenase (*Gapdh*) was used for normalization of the RTqPCR data. The sequences of the primers used for PCR were obtained from those validated at the PrimerBank database (Spandidos et al., 2009) and are shown in Table S1.

### Guanylin and uroguanylin ELISA

8–10-wk-old mice were sacrificed by CO_2_ asphyxiation. Blood was collected from the heart and allowed to clot overnight at 4°C. The serum was separated from blood clot by centrifuging at 10,000 *g *for 10 min at 4°C and stored at −80°C. Guanylin and uroguanylin levels in serum were estimated by ELISA (MyBioSource; guanylin: MBS104342, uroguanylin: MBS7223503) according to the manufacturer’s protocol.

### IEC isolation and cGMP estimation

Mice were sacrificed by CO_2_ asphyxiation, and ∼5 cm of the colon and 10 cm of the terminal ileum were harvested, flushed in ice-cold HBSS, cut longitudinally open, submerged in IEC dissociation buffer containing 10 mM Hepes, 1 mM EDTA, 71.5 mM β-mercaptoethanol, and 500 µM 3-isobutyl-1-methylxanthine (IBMX) to inhibit phosphodiesterases, and stored on ice. The tissues were incubated at 37°C 100 rpm for 45 min and vortexed for 30 s, and the tissue pieces were removed gently. The tubes were centrifuged at 3,000 rpm for 10 min at 4°C. The pellet containing the IECs was washed twice with ice-cold PBS and finally resuspended in homogenization buffer containing 50 mM Hepes, pH 7.5, 100 mM NaCl, 5 mM EDTA, 1 mM dithiothreitol, 5 µg/ml soya bean trypsin inhibitor (SBTI), 5 µg/ml leupeptin, 5 µg/ml aprotinin, 2 mM PMSF, 10 mM sodium orthovanadate, 1 mM sodium pyrophosphate, 20 mM sodium fluoride, and 500 µM IBMX. The cells were lysed by sonication at 60 cycles, 60% amplitude for 10 pulses (each pulse, 5 s; IKA Labortechnik). The suspension was centrifuged at 12,000 *g* for 60 min. The pellet containing the membrane fraction was resuspended in a buffer containing 50 mM Hepes, pH 7.5, 20% glycerol, 5 µg/ml SBTI, 5 µg/ml leupeptin, 5 µg/ml aprotinin, 2 mM PMSF, and 1 mM sodium orthovanadate, and protein concentration was estimated. Membrane preparations were used for ^125^I-labeled ST_Y72F_ binding assay, as described earlier (Saha et al., 2009), and Western blot analysis.

For cGMP estimation, the isolated and intact cells prepared from ∼5 cm of the colon or 10 cm of the terminal ileum were resuspended in PBS, and an aliquot of the suspension of cells was taken for protein estimation by Bradford assay (Bradford, 1976). Cells were harvested by centrifugation, resuspended in 0.1 N HCl, and heated at 95°C for 5 min. The mixture was then centrifuged at 17,000 rcf for 10 min at 4°C. The supernatant was collected, and cGMP levels were estimated using a cGMP ELISA kit (Cayman Chemicals). Cyclic GMP levels were normalized to the amount of protein taken for the cGMP ELISA.

### Colonic extract preparation for Western blot analysis

8-wk-old male wild type or S839I mice were sacrificed by CO_2_ asphyxiation, and 3 cm of the distal colon was harvested and snap-frozen in liquid nitrogen. The frozen tissue was crushed to a powder in liquid nitrogen using a mortar and pestle. The powdered tissue was transferred to homogenization buffer containing 50 mM Hepes, pH 7.5, 100 mM NaCl, 5 mM EDTA, 1 mM dithiothreitol, 5 µg/ml SBTI, 5 µg/ml leupeptin, 5 µg/ml aprotinin, 2 mM phenylmethylsulfonyl fluoride, 10 mM sodium orthovanadate, 1 mM sodium pyrophosphate, and 20 mM sodium fluoride. The extract was then subjected to sonication at 60 cycles, 60% amplitude (each pulse, 5 s; IKA Labortechnik) followed by centrifugation at 12,000 *g* for 60 min. The pellet containing the membrane fraction was resuspended in a buffer containing 50 mM Hepes, pH 7.5, 20% glycerol, 5 µg/ml SBTI, 5 µg/ml leupeptin, 5 µg/ml aprotinin, 2 mM phenylmethylsulfonyl fluoride, and 1 mM sodium orthovanadate. The supernatant containing the cytosol was collected. Protein concentrations in membrane and cytosolic fractions were estimated by the modified Bradford assay (Bradford, 1976). Both membrane and cytosolic fractions were used for Western blot analysis, depending on the cellular localization of the antigen being tested.

### Western blot

Membrane or cytosolic proteins from HEK293E cells, mouse IECs, or colonic tissue extracts were resolved on polyacrylamide gels (SDS-PAGE) and transferred onto Immun-Blot polyvinylidene fluoride membrane (Bio-Rad) in transfer buffer (25 mM Tris base, 192 mM glycine, and 20% methanol, pH 8.3) at −160 V and 200 mA for 120 min. The polyvinylidene fluoride membranes were rinsed in 10 mM Tris-Cl, pH 7.2, 100 mM NaCl, and 0.1% Tween 20 (TBS-T) and blocked for 1 h at room temperature using 5% blocking solution (GE Healthcare) prepared in TBS-T. The membrane was incubated with indicated antibodies overnight (12–14 h) at 4°C followed by three washes with TBS-T. The membrane was then incubated with anti-mouse IgG (at 1:6,000 dilution) or anti-rabbit IgG (at 1:30,000 dilution) conjugated to horseradish peroxidase for 1 h at room temperature, followed by three washes with TBS-T. Immunoreactive bands were visualized by chemiluminescence detected using Immobilon reagent (Millipore) on a Chemidoc XRS+ (Bio-Rad) imaging system. Anti-villin and anti-Na^+^/K^+^ adenosine triphosphatase antibodies were used at a dilution of 1:5,000. Anti-pSTAT1 Ser 727, anti-pSTAT1 Tyr701, anti-STAT1, anti-TAP1, anti-ISG15, and anti–β-actin antibodies were used at a dilution of 1:1,000. Anti-Ido1 antibody was used at a dilution of 1:4,000. Anti-NHE3 antibody was used at a dilution of 1:2,000. Anti-PKGII antibody was used at a dilution of 1:2,000. The sources of all commercial antibodies are shown in Table S2.

GCC:4B11 monoclonal antibody was raised against the kinase homology domain of human GC-C and is available in the laboratory. The supernatant from cultured cells was used for Western Blot analysis at a dilution of 1:100.

### Fecal water content

Fresh fecal pellets were collected from 4–5-wk-old mice in preweighed tubes. Collection was performed three times over the course of a year on independent litters. Tubes were weighed and then subjected to lyophilization for 12–14 h. The weight of the tube containing dry feces was recorded, and percentage water content in the feces was calculated using the following formula:


$$\% water content=\left[\begin{array}{l} \left(weight of wet feces – weight of dry feces\right)\\ /weight of wet feces \end{array}\right]\times \mathrm{100}.$$

### Gut motility studies

The frequency of bowel movements was determined as previously described (De Palma et al., 2015). 4-wk-old mice were used, and the experiment was performed between 8:00 a.m. and 9:00 a.m. Mice were placed in fresh autoclaved cages without any bedding material, and the number of pellets passed in the first 10 min was recorded as the frequency of bowel movements. Experiments were performed three times over the course of a year on independent litters.

To determine total gut transit time, nonfasted mice (7–8 wk old) were gavaged with 200 µl of nonabsorbable marker dye (6% wt/vol of carmine red in filter-sterilized 0.5% methylcellulose). The mice were housed individually in clean cages without bedding material with access to food and water. The mice were checked for output at 15-min intervals. The time taken for the first fecal pellet with carmine red to be passed was recorded as the total gut transit time. Experiments were performed three times over the course of a year on independent litters.

Small intestinal transit rate and GE were estimated using the GC and GE parameters as previously described (Sobczak et al., 2014) with a few modifications. Briefly, 8–12-wk-old mice were fasted overnight with free access to water. On the day of the experiment, mice were weighed and orally gavaged with 200 µl of a filter-sterilized marker dye mixture (50 mg/100 ml phenol red in 0.5% methylcellulose). 30 min after marker dye administration, mice were sacrificed, the gastrointestinal tract was isolated and kept chilled to reduce further peristalsis, and dissection was conducted as fast as possible. For the GC analysis, the small intestine was measured and divided into 10 equal parts. The intestinal segments were transferred to a tube containing 1 ml of distilled water and homogenized such that the intestinal contents were released into the water. The tubes were vortexed gently and centrifuged at 3,000 *g* for 5 min. Following this, 250 µl of the supernatant was transferred to another fresh tube containing 250 µl of 1N NaOH, and color was allowed to develop. The intensity of color was calorimetrically measured at 560 nm using a spectrophotometer (Tecan Infinite M200 Pro; Tecan Switzerland). GC of the small intestine was calculated using the following formula:

GC = Σ [(% A per segment×segment number)/100],

with GC ranges from 1 (minimum motility) to 10 (maximum motility).

To determine the GE of mice, the stomach was dissected carefully, its contents were transferred to a tube containing 2 ml distilled water, and the mixture was vortexed gently followed by centrifugation at 3,000 *g* for 5 min. 500 µl of supernatant was transferred to another tube containing 500 µl of 1N NaOH to develop a maximum intensity of color. The intensity of color (200 µl) was measured at 560 nm using a spectrophotometer. The percentage of dye that was present in the small intestine out of the total dye present in the stomach and the small intestine was a reflection of GE.

To test linaclotide-mediated enhancement in the small bowel transit rate, GC and GE analyses were performed using the protocol described earlier (Bryant et al., 2010) with a few modifications. The mice were fasted overnight with unlimited access to water. Mice were weighed and administered 100 µg/kg of body weight linaclotide (Cayman Chemicals) prepared in 25 mM Tris-HCl, pH 7.5, orally. Mice were replaced in their cages for 10 min and then sacrificed, and GC and GE analyses were performed.

To determine the effect of Cftr(inh)-172 (Sigma-Aldrich) on small bowel transit rate, overnight fasted mice were orally gavaged with 200 µg of Cftr(inh)-172 prepared in 10% wt/vol D-α-Tocopherol polyethylene glycol 1000 succinate (TPGS). Control mice received the vehicle alone (Thiagarajah et al., 2004). Mice were returned to their cages for 3 h, after which GC and GE analyses were performed.

To determine the effect of the tenapanor (NHE3 inhibitor; MedChem Express) on small bowel motility, mice were fasted overnight and orally gavaged with 1 mg/kg tenapanor (McHugh et al., 2018) prepared in 10% TPGS. Control mice received the vehicle alone. Mice were returned to their cages for 2 h, after which the GC and GE analyses were performed.

### Measurements of intestinal luminal pH and sodium

6–8-wk-old mice were sacrificed, and the intestine was isolated. Approximately 2-cm pieces of ileum and colon were cut open longitudinally, and the luminal side was exposed using a pair of forceps. The luminal pH and sodium ion concentration were measured using a pH meter (LAQUAtwin pH11; Horiba) and sodium ion meter (LAQUAtwin Na-11; Horiba), respectively.

To determine fecal sodium ion concentrations, fresh feces were collected from mice in preweighed tubes and weighed and subjected to lyophilization. Double-distilled water was added to each sample (100 mg/ml), and the fecal pellets were homogenized using a Spinwin micro pestle (Tarsons). The samples were vortexed for 30 s, followed by centrifugation at 3,000 rcf for 5 min. Sodium ion in the supernatant was measured using a sodium ion meter (LAQUAtwin Na-11; Horiba) and normalized to the weight of the dry feces.

### DSS-induced colitis

Male wild type and mutant mice were cohoused at the time of weaning (21 d of age) and remained cohoused for the entire duration of the experiment. Cohousing was performed in multiple cages to account for cage effects. 21 d after cohousing, mice were weighed, and feces were collected for estimation of basal levels of fecal lipocalin. Mice were administered 2.5% DSS (MP Biomedicals) in drinking water for 6 d, followed by drinking water for 1 d. Mice were observed every day for disease signs such as weight loss, diarrhea, blood in feces, or rectal bleeding. Disease activity index (DAI) was calculated as described earlier (Kim et al., 2012) and defined below.

$$\begin{array}{l} DAI = loss of body weight + stool consistency +\\ rectal bleeding. \end{array}$$

Loss of body weight scores were 0: no loss; 1: 1–5%; 2: 6–10%; 3: 11–15%; and 4: 16 to >20%. Stool consistency scores were 0: normal; 2: loose stool; and 4: diarrhea. Rectal bleeding scores were 0: no blood; 2: visual pellet bleeding; and 4: gross bleeding, blood around anus.

Mice were sacrificed on day 8 of the treatment, and the colon was isolated. The length of the colon was measured, and a ∼1-cm distal piece was collected and fixed in 4% neutral buffered formalin. Tissue was then dehydrated by serial immersion in increasing concentrations of ethanol and finally in paraffin using an automated tissue processing system (Leica Biosystems). The tissue was embedded in paraffin wax, and 4-µm sections were prepared using a microtome (Leica Biosystems). Sections were dewaxed and rehydrated by serial immersion in decreasing concentrations of ethanol. Finally, the sections were stained with hematoxylin for 5 min, followed by eosin for 1 min as previously described (Majumdar et al., 2018). Sections were imaged using an Axio-Observer Z1 microscope (Carl Zeiss Microimaging). Histomorphological evaluation of intestinal inflammation was performed in sections from four mice (Erben et al., 2014).

Crypt depth was measured in untreated mice to determine whether altered IEC turnover could have contributed to the increased damage seen in S839I mice (Fig. S3).

### Fecal lipocalin estimation

Fecal pellets collected before and on day 4 of DSS treatment were subjected to lipocalin ELISA as described (Chassaing et al., 2012). Briefly, the fecal pellets were collected and suspended in PBS containing 0.1% Tween 20 (100 mg/ml) and vortexed for 20 min to get a homogeneous fecal suspension. These samples were then centrifuged for 10 min at 12,000 rcf and 4°C. Clear supernatants were collected and stored at −20°C until analysis. Lipocalin levels were estimated in the supernatants using a Lipocalin2 ELISA kit (R&D Biosystems) according to the manufacturer’s protocol.

### DNA extraction and analysis of the fecal microbiome

Extraction of DNA from the fecal pellets of wild type (*n* = 11; 8 female and 3 male) and S839I (*n* = 10; 7 female and 3 male) mice was performed using the Fast DNA SPIN kit for soil (MP Biomedicals) according to the manufacturer’s protocol, with four bead-beating periods of 40 s. DNA concentration was normalized to 10 ng/µl by dilution with DNA elution solution (MP Biomedicals) to produce a final volume of 20 µl. DNA samples were sent to Clevergene Biocorp Pvt Ltd for PCR amplification of V3-V4 regions of 16S rRNA genes and paired-end sequencing (2 × 300 bp) on the Illumina MiSeq platform. The V3-V4 hypervariable regions of the 16S rRNA genes were amplified using the 341F (5′-CCTACGGGNGGCWGCAG-3′) and 805R (5′-GACTACHVGGGTATCTAATCC-3′) primers (Klindworth et al., 2013).

The raw paired-end reads were obtained in fastq format from the Illumina MiSeq platform. Taxonomic abundance tables were generated from fastq files using the dada2 pipeline (v1.14.0) for paired-end reads (Callahan et al., 2016) in R (v3.6.1). Briefly, quality score plots of sequence reads were inspected to determine the drop-off points in quality of reads based on which of the forward and reverse reads were truncated at 270 and 230 positions, respectively. Primer sequences were removed by trimming the 22 bp from the left end of the raw reads. Chimeric sequences were removed by using the remove Chimera Denovo function with the pooled sample inference method. Taxonomy was assigned to the chimera-free amplicon sequence variants (ASVs) using Silva database (v138, downloaded from McLaren, 2020). Data were filtered to remove ASVs assigned as mitochondria, chloroplasts, or other nonbacterial kingdoms and ASVs with less than two frequencies in total (singletons) using the phyloseq R package (McMurdie and Holmes, 2013; v1.30.0). Beta diversity measures were visualized using the principal coordinate analysis plot based on Bray-Curtis dissimilarity using the microbiome R package (Lahti et al., 2017; v1.8.0). Relative abundance at taxonomic levels of phyla and genera were based on ASV counts normalized as percentages (100 * [×/sum(×)]). Significance of differences between Shannon diversity index was determined using Wilcoxon rank sum test (P < 0.05) in the microbiome R package, while significant difference in relative abundance of taxa between wild type and S839I mice was tested using the Kruskal–Wallis test in STAMP statistical software (Parks et al., 2014).

Prediction of the functional content of gut microbiome from 16S rRNA gene ASV count dataset and representative sequence of each ASV was performed using the Piphillin tool (Narayan et al., 2020). In brief, this tool predicts functional attributes of microbial assemblages via direct nearest-neighbor matching between 16S rRNA gene amplicons and genomes from reference databases. Prediction was executed at 97% ID cutoff using KEGG (May 2020 release) and BioCyc22.5 reference databases. The output from Piphillin was analyzed by STAMP statistical software, using nonparametric Kruskal–Wallis test with Tukey-Kramer post hoc test (Parks et al., 2014).

### Transcriptomic analysis of murine colon

Approximately 1 cm of the distal colon was collected from four 8-wk-old female wild type and S839I mice in 1 ml of TRI Reagent (RNAiso Plus; TaKaRa) and snap-frozen in liquid nitrogen. RNA was isolated using QIAGEN RNeasy Mini kit according to the manufacturer’s protocol. RNA quality was assessed using agarose gel electrophoresis, and concentration of RNA was estimated using NanoDrop. 3 µg RNA was used for RNA-seq. The raw sequence data were generated using Illumina HiSeq by Clevergene Biocorp Pvt Ltd. Data quality of the raw reads was checked for base call quality distribution, Phred score, %GC, and sequencing adapter contamination using FastQC software. The samples that passed QC threshold (Q30 > 90%) were processed to remove adapter sequences and low-quality bases using fastp (Chen et al., 2018). Thereafter, the reads with Q30 > 90% were aligned with the indexed Mouse reference genome (GRCm 38.90; mm10) using STAR v2 aligner (Dobin et al., 2013). The PCR and optical duplicates from the aligned reads were removed using Picard tools. The aligned reads were then processed to obtain the gene level expression as read counts using featureCounts software (Liao et al., 2014). The eight samples were grouped as wild type and *Gucy2c^S839I^*, having four biological replicates for each group. Differential expression analysis between the two groups was performed using edgeR algorithm (Robinson et al., 2010) after normalizing the read counts based on trimmed-mean normalizing values. After normalization, 30,617 genomic features (58.17%) were removed from the analysis since they did not have at least one count per million in at least four samples. The resulting P values were adjusted using the Benjamini and Hochberg’s statistical test to control the FDR.

Analysis of data was performed using IPA (Qiagen) using log_2_ FC values of −2 and +1.5 and FDR < 0.05. Pathway analysis was performed with default settings, and z-scores for Upstream Regulators were provided by IPA. Analysis Match was used to identify gene sets with gene expression changes like those seen in our dataset, with a focus on datasets from DSS-treated mice and human UC patients.

### Data analysis

All values shown in the graphs represent the mean ± SD. Data analysis was performed using GraphPad Prism 9.2, and statistical analyses used for individual experiments are mentioned in the figure legends.

### Online supplemental material

Fig. S1 shows the creation and genotyping of the *Gucy2c*^S839I^ mice. Fig. S2 A shows the weight gain of wild type and S839I mice after parturition. Other parameters such as food and water intake, fecal weight, and urine output measured in male and female mice are also shown in Fig. S2 B. Fig. S3 A shows the levels of guanylin and uroguanylin in the sera of wild type and S839I mice. Fig. S3 B shows a Western blot of PKGII in the ileum and colon of wild type and S839I mice. Fig. S3 C shows colonic crypt depth measured from histological sections prepared from wild type and S839I mice. Table S1 shows the primers used in this study. Table S2 lists the antibodies used for Western blotting. Table S3 presents the KEGG pathway abundance data from the microbiome of S839I mice.

## Supplementary Material

Table S1 lists primers used in the study.

Table S2 shows antibodies used in the study.

Table S3 presents KEGG pathway abundance (P* < *0.005).

## Data Availability

RNA sequencing data have been deposited in ArrayExpress with accession no. E-MTAB-9148.
