# Correction: Gut-associated cGMP mediates colitis and dysbiosis in a mouse model of an activating mutation in *GUCY2C*

**DOI:** 10.1084/jem.2021047909292021c

**Published:** 2021-10-06

**Authors:** Vishwas Mishra, Avipsa Bose, Shashi Kiran, Sanghita Banerjee, Idrees A. Shah, Pooja Chaukimath, Mudasir M. Reshi, Swarna Srinivas, Anaxee Barman, Sandhya S. Visweswariah

Vol. 218, No. 11 | 10.1084/jem.20210479 | September 21, 2021

The authors regret that the legends to Fig. 3 (C, D, and I) and Fig. 4 (B, C, and E) did not originally specify that the values shown are adjusted P values. In addition, the colors used in the bar graph in Fig. 4 B were not correct nor defined. The corrected legends, with new text underlined, and corrected Fig. 4 are shown here.

The errors appear only in PDFs downloaded before September 27, 2021.

**Figure 3.**
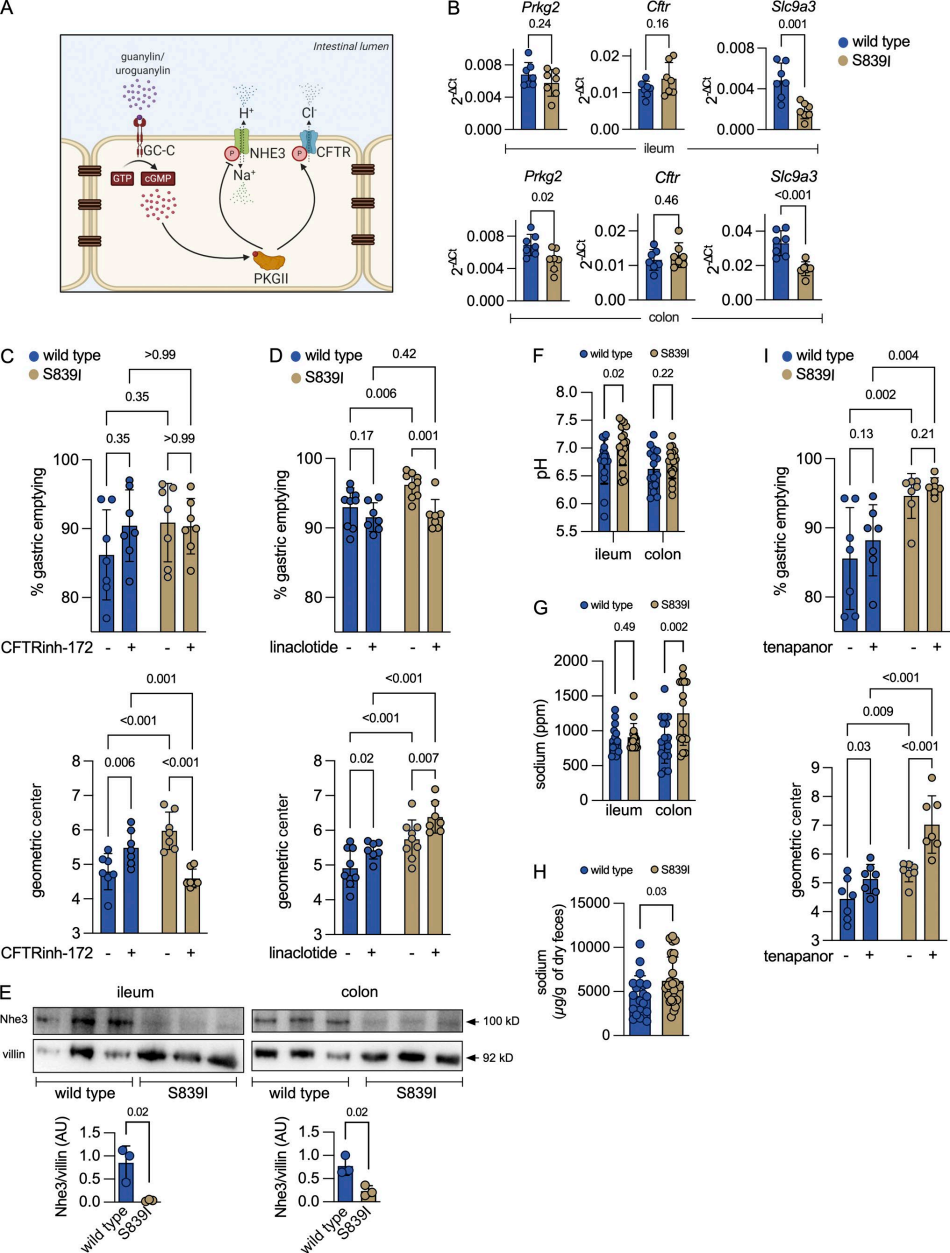
Elevated cGMP levels activate CFTR and inhibit Nhe3 in vivo. **(A)** Signaling events regulated by GC-C in the IEC. Phosphorylation of CFTR by PKGII increases chloride and bicarbonate secretion. Phosphorylation of NHE3 inhibits Na^+^ uptake. Image created using BioRender. **(B)** Transcript levels of genes downstream of GC-C. Each value represents data from an individual mouse, and the mean ± SD is shown. P values obtained from individual unpaired, two-tailed *t* tests with Welch’s correction are shown. Data are from seven female mice across two experiments. **(C)** GE and small intestinal transit after oral gavage with vehicle (TPGS) or CFTR(inh)-172. Values shown are from individual mice, and the mean ± SD is indicated. Two-way ANOVA with a two-stage linear step-up procedure of Benjamini, Krieger, and Yekutieli was performed, and adjusted P values are shown. Data are from experiments performed twice with a total of eight male and eight female mice of each genotype. **(D)** GE and small intestinal transit after oral gavage with buffer or linaclotide. Values shown are from individual mice, and the mean ± SD is indicated. Two-way ANOVA with a two-stage linear step-up procedure of Benjamini, Krieger, and Yekutieli was performed, and adjusted P values are shown. Data are from experiments performed twice with a total of eight male and eight female mice of each genotype. **(E)** Western blot showing Nhe3 expression in the membrane fraction of ileal (left) and colonic (right) epithelial cells. For samples from the ileum, 150 μg of protein was loaded, and for the colon, 80 μg of protein was loaded. The Nhe3 levels were normalized to villin. Graphs shown below depict densitometric analysis and represent the mean ± SD with data analyzed by an unpaired, two-tailed *t* test. Data are representative of three female mice. **(F)** Luminal pH in the ileum and the colon. Values shown are mean ± SD with data from individual mice, and P values shown are from an unpaired, two-tailed *t* test with Welch’s correction. Data are shown from 17 male and 9 female mice of both genotypes across experiments performed thrice. **(G)** Luminal sodium levels in the ileum and the colon. Values shown are mean ± SD with data from individual mice, and P values shown are from an unpaired, two-tailed *t* test with Welch’s correction. Data are from eight male and nine female mice, and experiments were performed twice. **(H)** Sodium content in fecal pellets collected. Values shown are the mean ± SD with data shown from individual mice, and P values shown are from an unpaired, two-tailed *t* test with Welch’s correction. Data from wild type mice are from 9 male and 12 female mice and from 12 male and 13 female S839I mice across experiments performed thrice. **(I)** GE and small intestinal transit after oral gavage with vehicle (TPGS) or the Nhe3 inhibitor (tenapanor). Values shown are from individual mice, and the mean ± SD is indicated. Two-way ANOVA with a two-stage linear step-up procedure of Benjamini, Krieger, and Yekutieli was performed, and adjusted P values are shown. Experiments were performed twice with a total of eight male and six female mice of each genotype.

**Figure 4.**
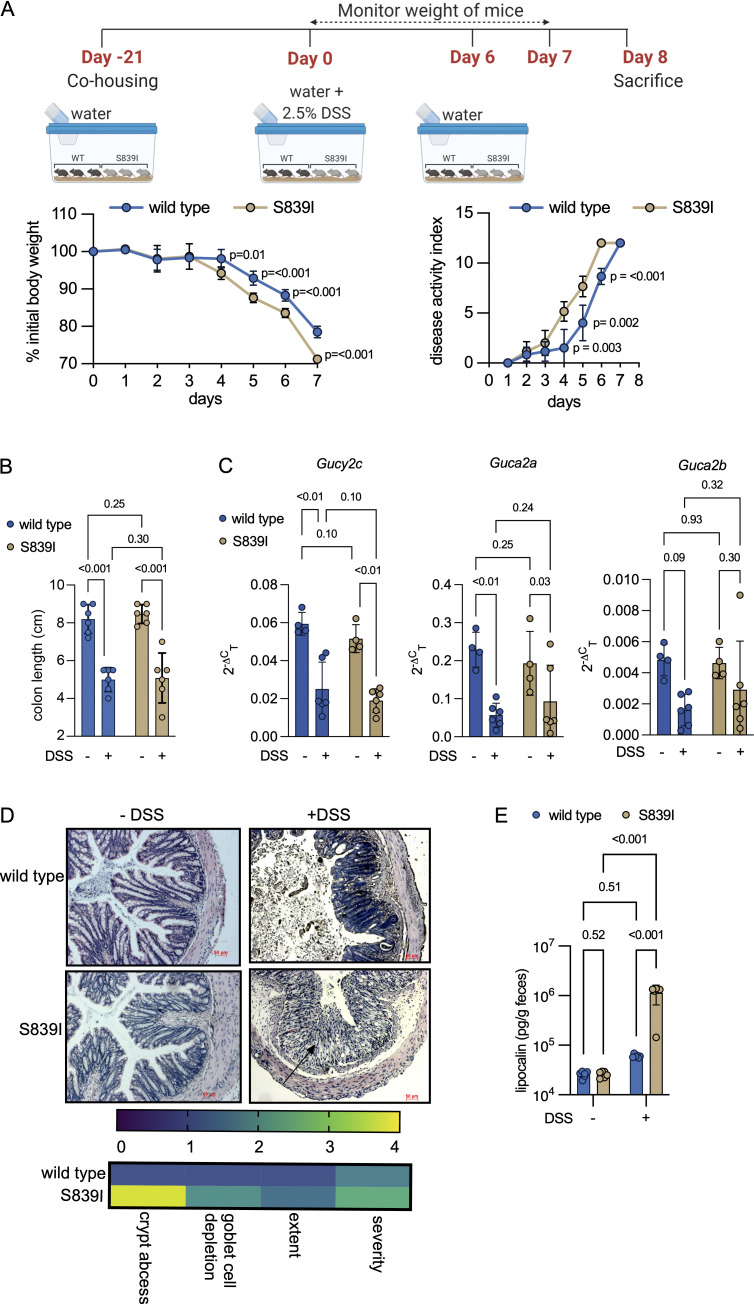
Mutant S839I mice show enhanced susceptibility to chemical-induced colitis. **(A)** Top: Depicts the details of DSS administration and monitoring of mice following induction of colitis. Left: Graph shows the mean weight loss observed following DSS exposure (six mice each). P values shown are from unpaired, two-tailed *t* tests with Welch’s correction at indicated time points. The experiment was repeated thrice, and data shown are from a single experiment and are the mean ± SD. Right: The disease activity index was scored, and P values are from unpaired, two-tailed *t* tests with Welch’s correction at indicated time points from the experiment shown in A. **(B)** Mice were treated with DSS or water and sacrificed on day 7. Each dot represents the colon length of an individual male mouse (six in total) across two experiments. Data shown are the mean ± SD and were analyzed by two-way ANOVA with a two-stage linear step-up procedure of Benjamini, Krieger, and Yekutieli, and adjusted P values are shown. **(C)** RTqPCR analysis of *Gucy2c* and its ligands following DSS treatment. Values shown are from individual male mice from a single experiment. The mean ± SD is shown. Data were analyzed by two-way ANOVA with a two-stage linear step-up procedure of Benjamini, Krieger, and Yekutieli, and adjusted P values are shown. Experiments were repeated thrice. **(D)** Sections were prepared from the distal colon of untreated or DSS-treated mice. Sections shown are representative of data from four mice, and the arrow indicates significant infiltration of immune cells into the colonic tissue in S839I mice. Scale bars, 50 µm. Images were evaluated for histomorphological changes. The mean across four wild type and four S839I mice is shown in the heat map. **(E)** Fresh feces were collected from each mouse before and 4 d after DSS treatment. Fecal lipocalin levels were estimated using ELISA. Values shown are the mean ± SD from six individual male mice. Data shown are the mean ± SD and were analyzed by two-way ANOVA with a two-stage linear step-up procedure of Benjamini, Krieger, and Yekutieli, and adjusted P values are shown. Experiments were repeated twice.

