# Supplementary material for: Gut-associated cGMP mediates colitis and dysbiosis in a mouse model of an activating mutation in GUCY2C
Source: J Exp Med. 2021 Sep 21;218(11):e20210479. doi: 10.1084/jem.20210479 (PMC8480670; doi:10.1084/jem.20210479)
Supplement: Table S1 — lists primers used in the study. [file JEM_20210479_TableS1.docx]

**Supplemental Table 1. Primers used in the study**

| **Primer name** | **Sequence (5ʹ–3ʹ)** |
| --- | --- |
| m*Gucy2c*_RT_fwd | AACCGCAGATGAGAAGGAGC |
| m*Gucy2c*_RT_rvs | CGTCGGATCAAGGTGTCCAT |
| m*Guca2a*_RT_fwd | GTTTGCTCTCAGGCTTCCGA |
| m*Guca2a*_RT_rvs | CAGGCAGCATAGGCACAGAT |
| m*Guca2b*_RT_fwd | CTCCAGCCTGTTTGTGCATC |
| m*Guca2b*_RT_rvs | AAGTATGGGCAGGGTAGGCT |
| m*Gapdh*_RT_fwd | CAACTCCCTCAAGATTGTCAGCAA |
| m*Gapdh*_RT_rvs | GGCATGGACTGTGGTCATGA |
| m*Slc9a3*_RT_fwd | CCGCCTCAGCAACAAATCAG |
| *mSlc9a3*_RT_rvs | AGCGTCCAGTAAGTGGTGTG |
| m*Ido1*_RT_fwd | GCTTTGCTCTACCACATCCAC |
| m*Ido1*_RT_rvs | CAGGCGCTGTAACCTGTGT |
| m*Cftr*_RT_fwd | GCACACTGAACATCACCGAAG |
| m*Cftr*_RT_rvs | GCACCAAATCAGCACTGCAA |
| m*Prkg2*_RT_fwd | GGTGGAATGCATGTACGGGA |
| m*Prkg2*_RT_rvs | CCACATTGGGATGGACGACA |
| m*Ifit1*_RT_fwd | AACCCAGAGAACAGCTACCACC |
| m*Ifit1*_RT_rvs | CAAGGAACTGGACCTGCTCTGA |
| m*Ifitm3*_RT_fwd | GCCTACGCCTCCACTGCTAA |
| m*Ifitm3*_RT_rvs | GGACCGGAAGTCGGAATCCT |
| m*Isg15*_RT_fwd | GCGGGAACAAGTCCACGAAG |
| m*Isg15*_RT_rvs | CCTCAGGCGCAAATGCTTGA |
| m*Tap1*_RT_fwd | TGGCTGAAGTCTGGACCACG |
| m*Tap1*_RT_rvs | ATGAGACAAGGTTGCCGCTG |
| m*Ifit3*_RT_ fwd | TCAGCCCACACCCAGCTTTT |
| m*Ifit3*_RT_rvs | CCTGGTTGCACACCCTGTCT |
| m*Socs1*_RT_fwd | CTGCGGCTTCTATTGGGGAC |
| m*Socs1*_RT_rvs | AAAAGGCAGTCGAAGGTCTCG |
| m*Irf7*_RT_fwd | GAGACTGGCTATTGGGGGAG |
| m*Irf7*_RT_rvs | GACCGAAATGCTTCCAGGG |
| m*Stat1*_RT_fwd | TCACAGTGGTTCGAGCTTCAG |
| m*Stat1*_RT_rvs | GCAAACGAGACATCATAGGCA |
| m*Ifng*_RT_fwd | ATGAACGCTACACACTGCATC |
| m*Ifng*_RT_rvs | CCATCCTTTTGCCAGTTCCTC |
