# Supplementary material for: Gut-associated cGMP mediates colitis and dysbiosis in a mouse model of an activating mutation in GUCY2C
Source: J Exp Med. 2021 Sep 21;218(11):e20210479. doi: 10.1084/jem.20210479 (PMC8480670; doi:10.1084/jem.20210479)
Supplement: Table S2 — shows antibodies used in the study. [file JEM_20210479_TableS2.docx]

**Supplemental Table 2.** Antibodies used in the study

| **Antibodies** | **Source** |
| --- | --- |
| Anti-GCC (4B11) | Monoclonal, raised in the lab against KHD of human GCC |
| Anti-pSTAT1 (S727) | Cell Signalling Technology (Cat. No.: 8826S) |
| Anti-pSTAT1 (Y701) | Cell Signalling Technology (Cat. No.: 9167S) |
| Anti-STAT1 | Cell Signalling Technology (Cat. No.: 9172S) |
| Anti-villin | Abcam (Cat. No.: ab130751) |
| Anti-mouse IgG, HRP conjugated | Sigma-Aldrich (Cat. No.: A6782) |
| Anti-rabbit IgG, HRP conjugated | Sigma-Aldrich (Cat. No.: A0545) |
| Anti-Na^+^/K^+^ ATPase | Abcam (Cat. No.: ab76020) |
| Anti-TAP1 | Cell Signalling Technology (Cat. No.: 12341S) |
| Anti-ISG15 | Cell Signalling Technology (Cat. No.: 2743S) |
| Anti-β-actin | Cell Signalling Technology (Cat. No.: 4970S) |
| Anti-Ido1 | Adipogen (Cat. No.: AG-25A-0032) |
| Anti-NHE3 antibody | Novus Biotech (Cat. No.: NBP1-46581) |
| Anti PKGII antibodies | Invitrogen (Cat. No. PA5-50931) |
