# Supplementary figures and images for: Gut-associated cGMP mediates colitis and dysbiosis in a mouse model of an activating mutation in GUCY2C
Source: J Exp Med. 2021 Sep 21;218(11):e20210479. doi: 10.1084/jem.20210479 (PMC8480670; doi:10.1084/jem.20210479)

**Supplemental Table 3**


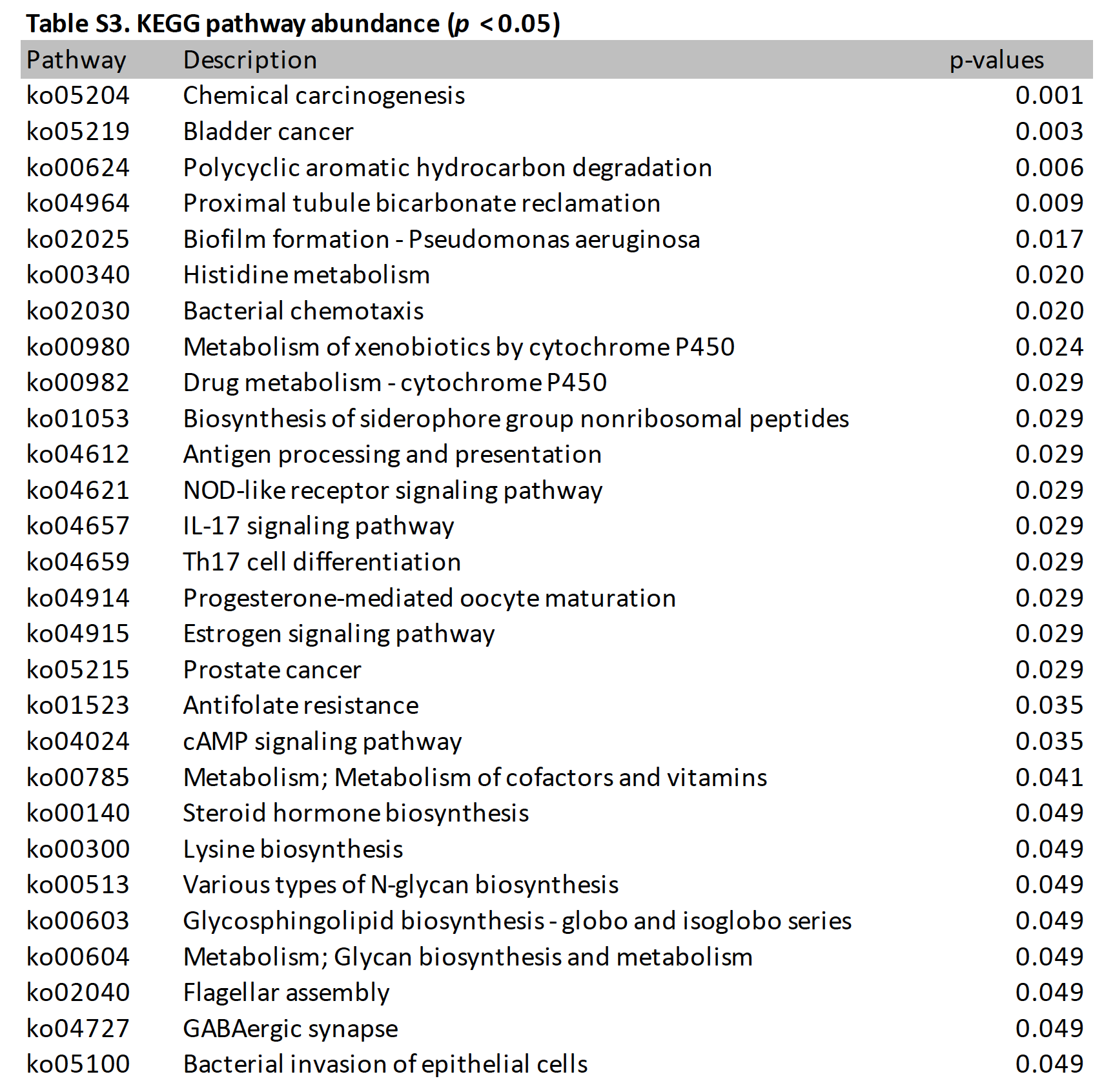

Supplement: Table S3 — presents KEGG pathway abundance (P < 0.005). [file JEM_20210479_TableS3.docx]
